# The Intestinal Barrier Protective Effect of Indole Aldehyde Derivatives on Acute *Toxoplasma gondii* Infection

**DOI:** 10.3390/molecules29215024

**Published:** 2024-10-24

**Authors:** Jieqiong Wang, Weifeng Yan, Xu Cheng, Yonggang Tong, Sihong Wang, Chunmei Jin

**Affiliations:** 1 Key Laboratory of Natural Medicines of the Changbai Mountain, Ministry of Education, College of Pharmacy, Yanbian University, Yanji 133002, China; jieqiong990804@163.com (J.W.); 2024001148@ybu.edu.cn (W.Y.); 0000007999@ybu.edu.cn (X.C.); 2023010901@ybu.edu.cn (Y.T.); 2 Analysis and Inspection Center, Yanbian University, Yanji 133002, China

**Keywords:** anti-*T. gondii*, indole derivatives, intestinal barrier protective effect, Vilsmeier–Haack reaction

## Abstract

Toxoplasmosis, a zoonotic infection caused by *Toxoplasma gondii* (*T. gondii*), poses a significant risk to human health and public safety. Despite the availability of clinical treatments, none effectively mitigate the intestinal barrier damage, which is the primary defense against *T. gondii* invasion. This study introduced aldehyde groups into the indole scaffold of a peptide-like structure to investigate the protective effects of these indole aldehyde derivatives on the intestinal barrier in mice with acute *T. gondii* infection. This approach leveraged the propensity of peptides and aldehyde groups to form hydrogen bonds. We synthesized a range of indole derivatives using the Vilsmeier–Haack reaction and evaluated their intestinal barrier protective effects both in vitro and in vivo. Our findings revealed that indole derivatives **A1** (1-Formyl-1*H*-indole-3-acetonitrile), **A3** (Indole-3-carboxaldehyde), **A5** (2-Chloro-1*H*-indole-3-carboxaldehyde), **A8** (1-Methyl-indole-3-carboxaldehyde), and **A9** (1-Methyl-2-phenyl-1*H*-indole-3-carboxaldehyde) demonstrated a higher selectivity index compared to the positive control, spiramycin. These derivatives enhanced gastrointestinal motility, increased glutathione (GSH) levels in the small intestine, and reduced malondialdehyde (MDA) and nitric oxide (NO) levels in the small intestine tissue and diamine oxidase (DAO) and NO levels in the serum of infected mice. Notably, **A3** exhibited comparable anti-*T. gondii* tachyzoites activity in the peritoneal cavity. Molecular docking studies indicated that the aldehyde group on the indole scaffold not only formed a hydrogen bond with NTPase-II but also interacted with TgCDPK1 through hydrogen bonding. Among the derivatives, **A3** showed promising intestinal barrier protective effects in mice with acute *T. gondii* infection. This research suggests that indole derivatives could serve as a potential therapeutic strategy for intestinal diseases induced by *T. gondii,* offering a novel direction for treating intestinal barrier damage and providing valuable insights for the chemical modification of drugs targeting *T. gondii*. Furthermore, it contributes to the advancement of therapeutic approaches for toxoplasmosis.

## 1. Introduction

Toxoplasmosis is a zoonic disease caused by *Toxoplasma gondii* (*T. gondii*). As a kind of classic obligate intracellular protozoan, *T. gondii* has a special life cycle and can infect most kinds of warm-blooded animals, so toxoplasmosis has been found all over the world [1,2]. Except for the usual transmission, like mother-to-infant transmission and blood-borne transmission, the most defenseless type of transmission is through the ingestion of *T. gondii*-contaminated food and water. In this way, the parasites enter the gastrointestinal system of hosts within a few days, rapidly cross the small intestinal epithelial barrier in just seconds, and then spread all over the organs [3]. *T. gondii* infection causes a retrograde migratory response in which large numbers of neutrophils migrate into the intestinal lumen to be infected, which then establishes new foci of infection throughout the intestinal tract [4]. After a period of time, the polarized Th1 immune response generated by the host leads to the overproduction of IFN-γ and Tregs, and, consequently, dysregulation of the Th1 response occurs, leading to irreversible pathological changes and inflammatory responses, which further exacerbate tissue damage and systemic inflammation [5]. Due to the severe inflammatory effect caused by *T. gondii*, many systemic diseases can occur in people, like encephalitis, neonatal malformations, chorioretinitis, and intellectual disability of the fetus, especially for patients with immunosuppression, organ transplantation, and AIDS, because the opportunistic pathogenicity of *T. gondii* occurs in this term [6]. The high death rate of these kinds of people is caused by intense organ damage and immunology response [7,8]. Although a proper inflammatory reaction is of benefit for eradicating pathogens, the inflammatory damage caused by *T. gondii* is usually uncontrolled [9]. As the first immune response organ during *T. gondii* infection, inflammation in the small intestine is especially severe [10]. This situation leads to disruption of the gut microbiota and exacerbates the damage to intestinal and brain barriers [11].

Unfortunately, the current clinical treatment for *T. gondii* infection is the use of sulfonamide and pyrimethamine combination, and only spiramycin (Spy) is permitted by the NMPA (National Medical Products Administration in China) for treatment in maternity. Sulfonamide and pyrimethamine show effects only in acute infection and are accompanied by high cytotoxicity; other drugs like macrolides have also been questioned due to drug resistance [12,13]. Finding a kind of compound with high effects and low toxicity to treat *T. gondii* infection and develop medicines is an emergency issue.

In the past few years, we have worked on screening anti-*T. gondii* compounds and have found some natural products with a basic nucleus. These compounds always showed low cytotoxicity with ample H-bonds. In a recent study, we noticed that intestine damage caused by *T. gondii* was relevant to the survival rate of mice, so we focused on finding a series of compounds with protective effects for the intestine that met the indole. In living systems, indoles are present in the essential amino acid tryptophan, the neurotransmitter 5-hydroxytryptamine, and the neurohormone melanin. Of these, tryptophan may play a biological role through the tryptophan-derived intestinal bacterial product, indoles, including indoleacetic acid, indole ethanol, indole-3-propionic acid (IPA), and others. These metabolites can directly regulate the expression of goblet cell secretion products to improve mucosal barrier integrity and also protect intestinal barrier function by regulating TJ, mucin production, and goblet cell secretion products [14]. Another piece of evidence that indoles are associated with intestinal inflammation and intestinal barriers is that in human intestinal epithelial cells, indoles and 7-hydroxyindoles increase intestinal epithelial cell tight junction resistance and decrease inflammatory cytokine levels [15]. Oral administration of indoles also increased the expression of tight and adherent junctions in the colon in a sterile mouse model [16]. Indole-3-lactic acid (ILA) could activate the aromatic hydrocarbon receptor signaling pathway in intestinal epithelial cells, promoting the activation of CD8(+) T cells and the secretion of interferon-gamma [11].

Tryptophan is an essential amino acid for *T. gondii* and plays an irreplaceable role in its intracellular proliferation [17,18]. Tryptophan is characterized by its indole functional group, and some compounds including indoles could be tryptophan-competing molecules, showing a good transport mechanism for indole compounds in anti-*T. gondii* activity [19,20,21]. The first investigation of the anti-*T. gondii* mechanism showed that 2-Phenylthioindole exhibited inhibitory effects on NTPase, which is a necessary enzyme for the rapid proliferation of tachyzoites in parasitic vesicles, such as *T. gondii*, but the specific mechanism of action was not clear [22,23]. However, the N-H structure of indole gives compounds a good ability to form hydrogen bonds with protons, which was one of the factors enabling the low cytotoxicity of indole compounds [24]. In summary, indole derivatives not only inhibit the activity of NTPase enzymes required for *T. gondii* replication in host cells but also interfere with absorption and metabolic processes of the essential amino acid tryptophan. In addition, indoles and indole-producing bacteria had beneficial effects in establishing the intestinal epithelial barrier and preventing intestinal inflammation. These characteristics of indole derivatives showed potential applications in anti-*T. gondii* activity and intestinal barrier protection in *T. gondii* infection [25]. We also noticed that aldehyde groups readily form hydrated aldehydes in biological microenvironments, which could increase the opportunity for compounds to form hydrogen bonds [26,27]. N-benzyl indole aldehydes had been reported as having better ability to scavenge antioxidants and regulate NO level in vitro [28] and some compounds had an aldehyde group, while two possessed a cyanohydrin group, with the alpha-bourbonene skeleton showing anti-fungal activity [29]. Incorporating a fragment of aldehyde into 4-thiazolidinone’s ring together with sulfanilamide pharmacophore could also significantly increase the antibacterial activity, and there was much evidence revealing the activity of aldehyde groups. It is worth noting the anti-parasite effect of indole and their derivatives.

So, enhancing the anti-*T. gondii* activity in vitro and in vivo of indole derivatives by introducing aldehyde groups using the Vilsmeier–Haack reaction was selected as the research object to synthesize a series of indole derivatives. Our study focuses on their role in intestinal barrier protection under *T. gondii* infection.

## 2. Results and Discussion

### 2.1. The Synthesis and Characterization of Indole Derivatives

#### 2.1.1. The Characterization of Indole Derivatives 1-Formyl-1*H*-indole-3-acetonitrile (**A1**)

Pale yellow solid (Rf = 0.55, yield 108.6 mg, 92.19%). In the ^1^H NMR (300 MHz, CDCl_3_) spectrum, there was an aldehyde hydrogen signal [*δ* 9.50 (s), 9.10 (s), 1H], three hydrogen signals on the indole ring *δ* 8.40 (d, *J* = 6.0 Hz, 1H), 7.75 (d, *J* = 37.3 Hz, 1H), 7.55–7.32 (m, 3H), and one hydrogen signal on the methylene group *δ* 3.79 (d, *J* = 1.1 Hz, 2H). Signal *δ* 3.79 (d, *J* = 1.1 Hz, 2H). In ^13^C NMR (75 MHz, CDCl_3_), there is an aldehyde carbon signal *δ* 197.8, seven carbon signals on the indole ring *δ* 159.1, 155.7, 129.2, 126.3, 125.3, 124.3, 118.4, 116.8, a cyano carbon signal *δ* 116.4, a methylene carbon signal *δ* 14.5.

Upon comparing the ^1^H NMR spectra of 3-indoleacetonitrile before and after the Vilsmeier–Haack reaction, a new proton signal was observed at a chemical shift of δ 9.40. This signal corresponded to the aldehyde proton of the formylated 3-indoleacetonitrile, indicating the replacement of the hydrogen on the nitrogen atom. In summary, the spectroscopic data of the product aligns with the literature [30], confirming its identification as 1-formyl-1*H*-indole-3-acetonitrile.

#### 2.1.2. The Characterization of Indole Derivatives 1-Formyl-1*H*-indole-3-ethanamine (**A2**)

Pale yellow solid (Rf = 0.28, yield 22.0 mg, 48.74%). In the ^1^H NMR (300 MHz, CDCl_3_) spectrum, there was one aldehyde group hydrogen signal *δ* 8.13 (s, 1H), three indole ring hydrogen signals *δ* 7.61 (d, *J* = 7.8 Hz, 1H), 7.39 (d, *J* = 8.1 Hz, 1H), 7.24–7.01 (m, 3H), and two methylene group hydrogen signals *δ* 3.66 (t, *J* = 6.5 Hz, 2H), *δ* 3.00 (t, *J* = 6.5 Hz, 2H) and *δ* 3.00 (t, *J* = 6.5 Hz, 2H). Hz, 2H), *δ* 3.00 (t, *J* = 6.5 Hz, 2H), one amino group hydrogen signal *δ* 1.62 (s, 2H). In ^13^C NMR (75 MHz, CDCl_3_), and there was one aldehyde carbon signal *δ* 161.1, eight carbon signals on the indole ring *δ* 136.4, 127.2, 122.3, 122.1, 119.6, 118.7, 112.6, 111.4, two methylene carbon signals *δ* 38.3, 25.2. In summary, the analysis confirmed that this product is consistent with the spectral data in the literature [31] and it is identified as 1-Formyl-1*H*-indole-3-ethanamine.

#### 2.1.3. The Characterization of Indole Derivatives 1-Formyl-1*H*-indole-3-ethanamine (**A3**)

Off-white crystals (R_f_ = 0.61, yield 50.1 mg, 80.33%). In the ^1^H NMR (300 MHz, CDCl_3_) pattern, there were hydrogen signals *δ* 10.08 (s, 1H) on one aldehyde group, *δ* 8.81 (s, 1H) on one nitrogen atom, *δ* 8.32 (dd, *J* = 8.3, 4.9 Hz, 1H) on four indole rings, 7.86 (d, *J* = 3.1 Hz, 1H), 7.45 (dt, *J* = 4.9, 2.9 Hz, 1H), 7.37–7.29 (m, 2H). In ^13^C NMR (75 MHz, CDCl_3_), there is a carbon signal *δ* 185.2 for one aldehyde group, and the rest belong to carbon signals *δ* 135.2, 135.2, 124.5, 123.1, 123.1, 122.0, 122.0, 111.5 on the indole ring. The above analysis confirms that this product is indole-3-carbaldehyde, in agreement with the literature spectral data [32].

#### 2.1.4. The Characterization of Indole Derivatives 5-Bromo-1*H*-pyrrolo[2,3-b] Pyridine-3-carbaldehyde (**A4**)

Pale yellow solid (R_f_ = 0.18, yield 10.1 mg, 22.55%). In the ^1^H NMR (300 MHz, CDCl_3_) spectrum, there was an aldehyde hydrogen signal *δ* 10.02 (s, 1H), a hydrogen signal on a nitrogen atom *δ* 9.90 (s, 1H), and hydrogen signals on the pyrrolidinium ring *δ* 8.77 (d, *J* = 2.0 Hz, 1H), 8.48 (d, *J* = 1.8 Hz, 1H), 7.99 (d, *J* = 2.6 Hz, 1H). In ^13^C NMR (75 MHz, CDCl_3_), there is one aldehyde carbon signal *δ* 184.8, and the rest belong to the carbon signals *δ* 155.7, 146.3, 135.8, 133.2, 125.6, 123.2, 115.2 on the pyrrolidinium ring. In summary, the analysis confirmed that this product is consistent with the spectral data in the literature [33] and it is identified as 5-bromo-1*H*-pyrrolo[2,3-b] pyridine-3-carbaldehyde.

#### 2.1.5. The Characterization of Indole Derivatives 2-Chloro-1*H*-indole-3-carbaldehyde (**A5**)

Pale yellow solid (R_f_ = 0.41, yield 22.7 mg, 93.97%). In the ^1^H NMR (300 MHz, CDCl_3_) spectrum, there was an aldehyde group hydrogen signal *δ* 10.14 (s, 1H), a hydrogen signal on a nitrogen atom *δ* 8.69 (s, 1H), and hydrogen signals on two indole rings *δ* 8.33–8.22 (m, 1H), 7.37–7.27 (m, 3H). In ^13^C NMR (75 MHz, CDCl_3_), there was one aldehyde carbon signal *δ* 183.4, and the rest belonged to carbon signals *δ* 134.8, 134.6, 124.3, 123.9, 122.8, 119.9, 112.0, 111.8 on the indole ring. In summary, the analysis confirmed that this product was consistent with the spectral data in the literature [34] and it is identified as 2-chloro-1*H*-indole-3-carbaldehyde.

#### 2.1.6. The Characterization of Indole Derivatives 1-Formyl-1*H*-indole-3-carboxylic Acid Methyl Ester (**A6**)

Pale yellow solid (R_f_ = 0.73, yield 9.1 mg, 17.93%). In the ^1^H NMR (300 MHz, CDCl_3_) spectrum, there was one aldehyde hydrogen signal *δ* 10.21 (s, 1H), four hydrogen signals on the indole ring *δ* 8.18 (dd, *J* = 5.6, 3.5 Hz, 1H), 7.91 (d, *J* = 2.9 Hz, 1H), 7.46–7.36 (m, 1H), 7.32–7.12 (m, 2H), a methyl hydrogen signal *δ* 3.91 (s, 3H). In ^13^C NMR (75 MHz, CDCl_3_), there was one aldehyde carbon signal *δ* 166.1, one carbonyl carbon signal *δ* 162.8, one methyl carbon signal *δ* 51.0 (s), and the rest belonged to carbon signals on the indole ring *δ* 136.5, 131.7, 125.9, 122.8, 121.7, 121.2, 111.9, 107.9. MS (EI) *m*/*z*: 226.99 ([M + Na]^+^, 24%) for C_11_H_9_O_3_N_1_. In summary, the above analysis and literature search did not reveal any compound identical to it, and it was identified as 1-formyl-1*H*-indole-3-carboxylic acid methyl ester.

#### 2.1.7. The Characterization of Indole Derivatives 2-Chloro-1*H*-indole (**A7**)

Orange-red solid (R_f_ = 0.67, yield 21.6 mg, 95.35%). In the ^1^H NMR (300 MHz, CDCl_3_) spectrum, there is a hydrogen signal *δ* 8.05 (s, 1H) on one nitrogen atom and *δ* 7.52 (d, *J* = 7.7 Hz, 1H), 7.29 (d, *J* = 8.2 Hz, 1H), 7.20 (dd, *J* = 8.1, 0.8 Hz, 1H), 7.15 (dd, *J* = 7.7, 1.2 Hz, 1H), and 6.42 (d, *J* = 0.8 Hz, 1H) on five indole rings. In ^13^C NMR (75 MHz, CDCl_3_), *δ* 135.0, 128.1, 123.3, 122.3, 120.6, 119.9, 110.3, 100.8. In conclusion, the analysis confirmed that this product is consistent with the spectral data in the literature [35] and it is identified as 2-chloro-1*H*-indole.

#### 2.1.8. The Characterization of Indole Derivatives 1-Methyl-indole-3-carbaldehyde (**A8**)

Off-white crystals (Rf = 0.61, yield 141.6 mg, 95.3%). In the ^1^H NMR (300 MHz, DMSO-*d*6) pattern, there were hydrogen signals *δ* 9.91 (s, 1H) on one aldehyde group, *δ* 3.90 (s, 3H) on methylone of the nitrogen atom, 8.19 (s, 1H), 7.58 (d, *J* = 7.8 Hz, 1H), 7.42 (dd, *J* = 8.1, 0.8 Hz, 1H), 7.31 (dd, *J* = 7.6, 0.8 Hz 1H), 7.03(d, *J* = 7.9 Hz, 1H) on indole rings. In ^13^C NMR (75 MHz, DMSO-*d*6), there is a carbon signal *δ* 184.2 (s) for one aldehyde group, there is a carbon signal *δ* 141.6 (s) for methylone of the nitrogen atom, and the rest belong to carbon signals *δ* 137.7 (s), 124.6 (s), 123.5 (s), 122.5 (s), 120.9 (s), 117.0 (s), 111.0 (s), 33.3 (s) on the indole ring. The above analysis confirms that this product is 1-Methyl-indole-3-carbaldehyde in agreement with the literature spectral data [36].

#### 2.1.9. The Characterization of Indole Derivatives 1-Methyl-2-phenylindole-3-carbaldehyde (**A9**)

Off-white crystals (Rf = 0.43, yield 51.0 mg, 45%). In the ^1^H NMR (300 MHz, DMSO-*d*6) pattern, there were hydrogen signals *δ* 9.61 (s, 1H) on one aldehyde group, *δ* 3.70 (s, 3H) on methylone of the nitrogen atom, *δ* 8.24 (d, *J* = 7.2 Hz, 1H), 7.74–7.56 (m, 6H), 7.42–7.36 (m, 1H), 7.32 (d, *J* = 7.1 Hz, 1H) on indole rings and benzene. In ^13^C NMR (75 MHz, DMSO-*d*6), there is a carbon signal *δ* 185.2 (s) for one aldehyde group, there is a carbon signal *δ* 151.2 (s) for methylone of the nitrogen atom, and the rest belong to carbon signals *δ* 137.1 (s), 131.0 (s), 131.0 (s), 129.9 (s), 128.7 (s), 128.7 (s), 128.1 (s),124.5 (s), 123.8 (s), 123.0 (s), 120.9 (s), 114.4 (s), 111.0 (s), 31.1 (s), on the indole ring benzene. The above analysis confirms that this product is 1-Methyl-indole-3-carbaldehyde in agreement with the literature spectral data [37].

**Figure 1:**
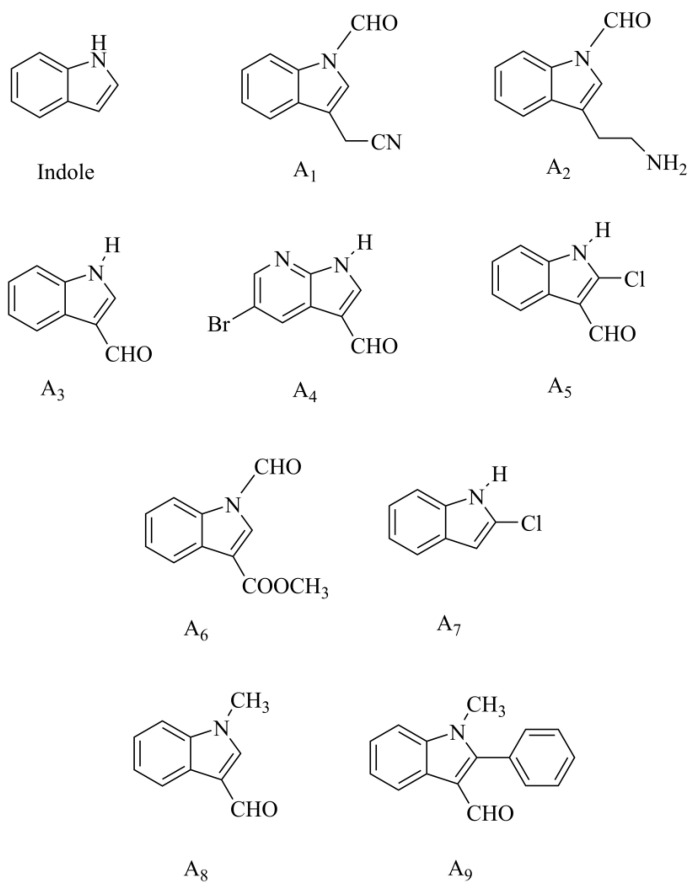
The chemical structures of compounds **A1**–**A9** involved in the study.

In a word, our research successfully synthesized nine indole-aldehyde derivatives, including one new compound, as depicted in Figure 1. The complete spectral data for these compounds are presented in the Supplementary Materials.

### 2.2. Effect of Indole Derivatives on Anti-T. gondii In Vitro

#### 2.2.1. Effect of Indole Derivatives on the Selectivity Index of *T. gondii*-Infected IEC-6 Cells and *T. gondii*

Anti-*T. gondii* activity was quantified using the selectivity index (SI), as presented in Table 1. The IC_50_ values in IEC-6 cells were as follows: 939.6 µM for indole, 416.7 µM for **A1**, 869.2 µM for **A3**, 960.3 µM for **A4**, 602.5 µM for **A5**, 632.7 µM for **A6**, 283.7 µM for **A7**, 649.9 µM for **A8**, 408.4 µM for **A9**, and 246.4 µM for Spy. All compounds exhibited low cytotoxicity in vitro. **A1** and **A3** demonstrated superior selective anti-*T. gondii* effects compared to the positive control, spiramycin, as indicated by their selectivity indices (**A1**: 1.2, **A3**: 1.3, Spy: 1.1). Compounds **A5**, **A8**, and **A9** exhibited anti-*T. gondii* effects in vitro comparable to that of Spy.

The IC_50_ value for **A2** exceeded 1000 µM, indicating no detectable anti-*T. gondii* activity within the tested concentration range. The underlying cause of this ineffectiveness is unclear but likely multifactorial. It may be related to the rapid metabolism of the ethanamine structure within the cell or its limited ability to traverse the phospholipid bilayer. Previous research suggests that incorporating an ethanamine structure into the benzimidazole skeleton reduces anti-*T. gondii* activity by inhibiting the interaction with *T. gondii* calcium-dependent protein kinase-1 (TgCDPK1)SpySpySpy [38].

#### 2.2.2. Effect of Indole Derivatives on Cellular Infectivity and Proliferation of *T. gondii*

**Figure 2:**
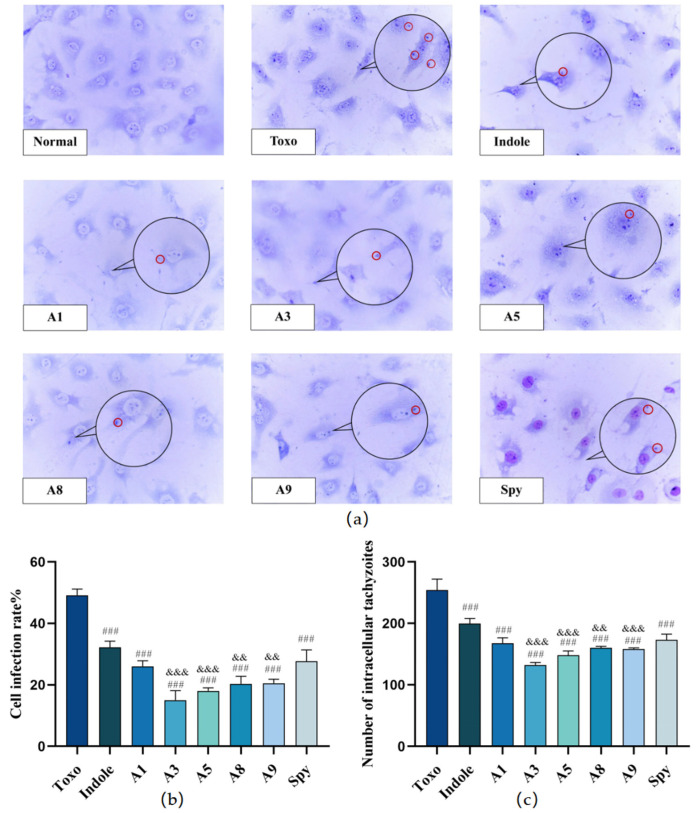
The effect of indole derivatives on *T. gondii* infection in vitro. (**a**) Anti-*T. gondii* activity of indole derivatives evaluated by Giemsa staining, and tachyzoites are marked by red circle (All images were in 40× field). (**b**) The effect of indole derivatives on cell infection rate with *T. gondii* infection. (**c**) The effect of indole derivatives on number of intracellular tachyzoites with *T. gondii* infection. Values are expressed as mean ± S.D (*n* = 6). ^###^ *p* < 0.001 compared with Toxo group; ^&&^ *p* < 0.01, ^&&&^ *p* < 0.001 compared with Spy group.

In this study, morphological alterations in intestinal epithelial cells post-*T. gondii* infection were examined using Giemsa staining to assess both the infection and intracellular proliferation of the pathogen. Figure 2b illustrates a decrease in the density of host cells following three hours of *T. gondii* exposure compared to the normal control group. Additionally, the invasion by *T. gondii* and subsequent formation of parasitized vacuoles were observed.

The infection rates in the Toxo and Spy groups were 49.2% with 254 intracellular tachyzoites and 27.7% with 173 intracellular tachyzoites, respectively. In comparison, treatment groups exhibited significant reductions in both cellular infection rates and numbers of intracellular tachyzoites (^###^ *p* < 0.001), with rates of 32.2% (Indole), 26.0% (**A1**), 15.0% (**A3**), 20.3% (**A5**), 20.5% (**A8**), 18.0% (**A9**), and 27.7% (Spy). Notably, compounds **A3**, **A5**, **A8**, and **A9** were more effective than Spy in decreasing both the host cell infection rate and the number of intracellular tachyzoites (^&&^ *p* < 0.01, ^&&&^ *p* < 0.001). Among them, **A3** demonstrated the most potent inhibitory effect on *T. gondii* infection and proliferation in vitro.

**Figure 3:**
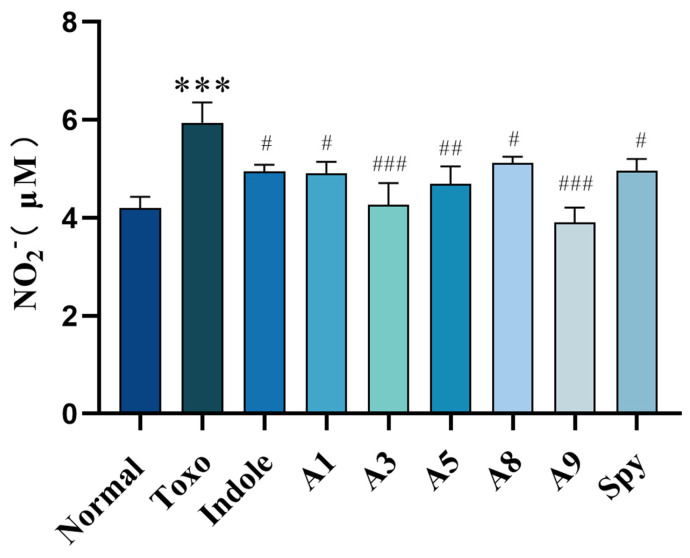
The effect of indole derivatives on NO level of IEC-6 cells with *T. gondii* infection. Values are expressed as mean ± S.D (*n* = 6). *** *p* < 0.001 compared with normal group; ^#^ *p* < 0.05, ^##^ *p* < 0.01, ^###^ *p* < 0.001 compared with Toxo group.

Furthermore, compounds **A3**, **A5**, **A8**, and **A9** appeared to specifically target *T. gondii*-infected IEC-6 cells, mitigating the infection. Protective effects on the integrity of IEC-6 cells under *T. gondii* infection were also noted, with cell integrity being well-preserved, as depicted in Figure 2a, underscoring the potential therapeutic benefits of these treatments. We assessed the nitric oxide (NO) levels in IEC-6 cells infected with *T. gondii*, as depicted in Figure 3, to evaluate intestinal damage due to inflammatory responses; NO is recognized as a key signaling molecule in inflammation [39]. *T. gondii* infection significantly increased NO release in host cells. (*** *p* < 0.001). Following the administration of indole derivatives, there was a significant reduction in NO levels compared to those in the Toxo group (^#^ *p* < 0.05, ^##^ *p* < 0.01, ^###^ *p* < 0.001). Specifically, treatments with derivatives **A3** and **A9** resulted in the lowest NO release levels. Thus, the indole derivatives significantly mitigated the increase in NO release induced by *T. gondii* infection in vitro. This suggests that derivatives **A3** and **A9** may alleviate the inflammatory response associated with *T. gondii* infection and potentially offer protective effects to host cells.

In summary, compounds **A1**, **A3**, **A5**, **A8**, and **A9** were effective in reducing the infection rate of host cells and significantly decreased the release of intracellular nitric oxide (NO) following infection with T. gondii. The inhibition of tachyzoites and the reduction in intracellular T. gondii by compounds **A3**, **A5**, **A8**, and **A9** surpassed the performance of the control drug Spy. Notably, **A3** and **A5** played a key role in the reduction in the release of NO.

### 2.3. Effect of Indole Derivatives on Anti-T. gondii In Vivo

#### 2.3.1. Effect of Indole Derivatives on Small Intestine Function of Mice with *T. gondii* Infection

**Figure 4:**
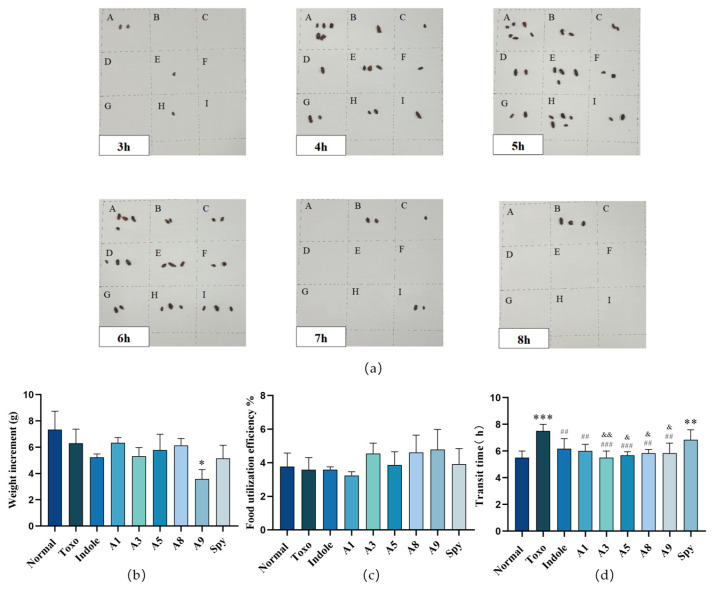
The protective effects of indole derivatives on small intestine function of mice with *T. gondii* infection in vivo. (**a**) Red pellet expulsion of mice, A (**normal**), B (**Toxo**), C (**Indole**), D (**A1**), E (**A3**), F (**A5**), G (**A8**), H (**A9**), I (**Spy**); (**b**) the weight increment of mice with *T. gondii* infection; (**c**) food utilization efficiency of mice with *T. gondii* infection; (**d**) carmine excretion time of mice with *T. gondii* infection. Values are expressed as mean ± S.D (*n* = 6). * *p* < 0.05, ** *p* < 0.01, *** *p* < 0.001 compared with normal group; ^##^ *p* < 0.01, ^###^ *p* < 0.001 compared with Toxo group; ^&^ *p* < 0.05, ^&&^ *p* < 0.01 compared with Spy group.

To evaluate the protective effects of indole derivatives on the small intestine of mice infected with *T. gondii*, we assessed gastrointestinal transport capacity alongside changes in body weight and food utilization efficiency during infection, as depicted in Figure 4. The reduction in body weight gain among *T. gondii*-infected mice was not significant compared to that of uninfected controls. Treatment with indole derivatives generally did not affect this parameter, except for derivative **A9**. Notably, mice treated with **A9** exhibited enhanced food utilization efficiency, which indicates a reduced food intake for this group. Mice infected with *T. gondii* exhibited a significantly prolonged duration for carmine excretion compared to uninfected controls, which was notably reduced following treatment with indole derivatives. As depicted in Figure 4a, uninfected mice demonstrated a consistent increase in the rate of pellet expulsion and an earlier emergence of red-colored pellets. Following *T. gondii* infection, the whole gut transit (WGT) time for carmine was significantly reduced. However, this impairment was mitigated by treatment with indole derivatives. WGT is considered a critical benchmark in preclinical studies for assessing gastrointestinal function [40], and has demonstrated a protective effect against *T. gondii* infection.

#### 2.3.2. Effect of Indole Derivatives on the Growth Inhibition of *T. gondii* In Vivo

The quantification of tachyzoites in the peritoneal cavities of mice infected with *T. gondii* was conducted, and the corresponding inhibition rates are presented in Table 2. Following the intraperitoneal injection of *T. gondii*, the tachyzoite count in the peritoneal cavity of the mice was observed to be 12.28 × 10^6^. Upon administration of various indole derivatives, significant changes in tachyzoite counts were noted, with inhibition rates of 22.3% for derivative **A1**, 33.3% for **A3**, 22.2% for **A5**, 18.3% for **A8**, and 28.1% for **A9**, respectively. Notably, derivative **A3** exhibited an anti-*T. gondii* effect in vivo comparable to that of Spy, yet demonstrated lower cytotoxicity. These findings underscore the need for further research into indole derivatives as potential therapeutic agents against *T. gondii*.

#### 2.3.3. Effect of Indole Derivatives on the Histology Study of Mice with *T. gondii* Infection In Vivo

**Figure 5:**
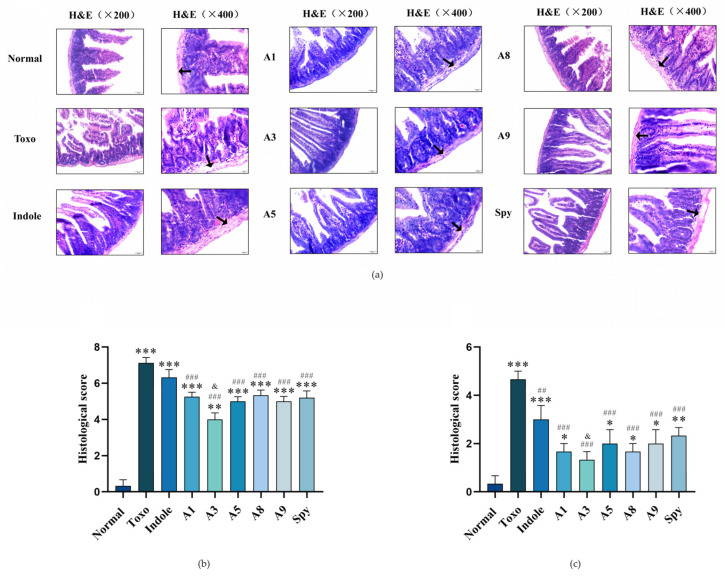
The protective effect histology study of indole derivatives on small intestine barrier of mice with *T. gondii* infection in vivo. (**a**) Histopathological photos of mice small intestine with H&E staining, and the barrier of small intestine is marked by arrows. (**b**) The mice intestine epigenetic pathology scoring of mice with *T. gondii* infection. (**c**) The mice intestine staining pathology score of mice with *T. gondii* infection. Values are expressed as mean ± S.D (*n =* 6). * *p* < 0.05, ** *p* < 0.01, *** *p* < 0.001 compared with normal group; ^##^ *p* < 0.01, ^###^ *p* < 0.001 compared with Toxo group; ^&^ *p* < 0.05 compared with Spy group.

The small intestine is the initial site of immune response in *T. gondii* infection in mice, exhibiting pronounced pathological alterations. Assessing these changes through pathological morphology provides a direct measure of damage to the small intestinal tissues. In this study, we evaluated the protective effects of indole derivatives on the intestinal barrier integrity in *T. gondii*-infected mice by conducting epigenetic pathology and histology scoring, as depicted in Figure 5. In uninfected control mice, the dissected small intestines were free from hemorrhages, edema, and residual digestive juices, displaying intact and normal tissues. Conversely, the *T. gondii*-infected group (Toxo group) showed significant hemorrhages and edema in the outer intestinal barrier, increased orange digestive juices, and instances of flatulence in the intestinal lumen. Upon treatment with indole derivatives, including Spy and other indole derivatives, no obvious differences in pathological scores were observed compared to the Toxo group. However, these treatments effectively mitigated histopathological manifestations by reducing blood filament presence in the outer intestinal barrier and alleviating edema. Although a small amount of orange digestive residue remained in the intestinal lumen of some mice, none of the treatments completely reversed the acute pathological damage caused by *T. gondii* infection. Notably, the control drug Spy showed no abnormalities in the small intestinal tissues. Among the treatments, administration of compound **A3** resulted in a significantly lower apparent pathological damage score compared to Spy, indicating a superior protective effect on the intestinal barrier.

In H&E staining, the intestinal villi of normal mice appeared structurally intact with mucosal epithelial cells closely arranged and clearly demarcated. The lamina propria of the small intestine was not thickened, and well-defined crypts were visible without any pathological alterations. Conversely, in *T. gondii*-infected mice, the small intestinal villi exhibited extensive shedding, and the arrangement of the villous epithelial cells was disrupted. Additionally, there was a noticeable reduction in the number of crypts, significant thickening of the lamina propria, and enlargement of the subepithelial space at the tips of the villi accompanied by marked inflammatory cell infiltration. These observations indicate severe tissue damage in the small intestines of *T. gondii*-infected mice, reflected by significantly elevated histopathological scores compared to those of normal mice.

Consequently, compounds **A1**, **A3**, **A5**, **A8**, and **A9** demonstrate potential in ameliorating intestinal barrier damage induced by *T. gondii* infection. Notably, the indole derivative **A3** was the most effective, surpassing the control drug Spy in efficacy.

#### 2.3.4. Effect of Indole Derivatives on the Small Intestine Biochemical Parameters of Mice with *T. gondii* In Vivo

**Figure 6:**
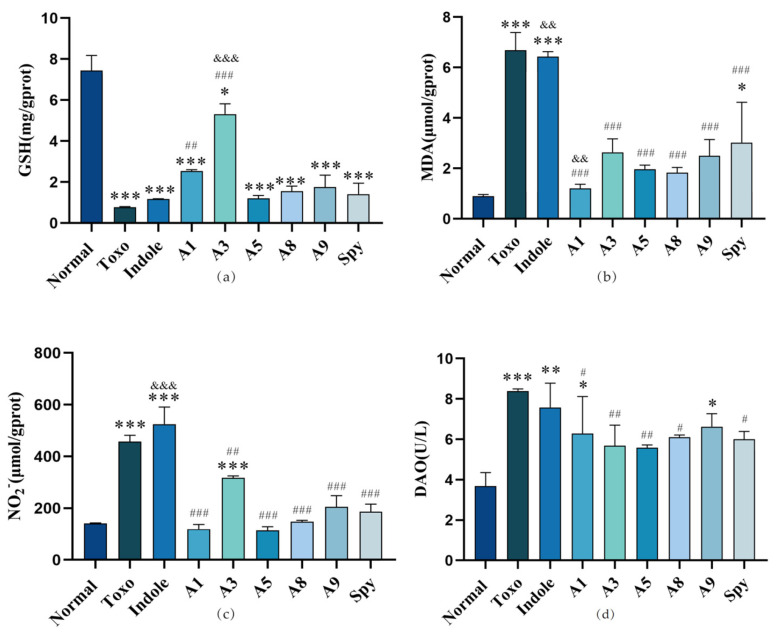
The effect of indole derivatives on small intestine biochemical parameters of mice with *T. gondii* in vivo: (**a**) GSH level in small intestine tissue; (**b**) MDA level in small intestine tissue; (**c**) NO level in small intestine tissue; (**d**) DAO level in serum. Values are expressed as mean ± S.D (*n =* 6). * *p* < 0.05, ** *p* < 0.01, *** *p* < 0.001 compared with normal group; ^#^ *p* < 0.05, ^##^ *p* < 0.01, ^###^ *p* < 0.001 compared with Toxo group; ^&&^ *p* < 0.01, ^&&&^ *p* < 0.001 compared with Spy group.

The primary factors contributing to intestinal injury include oxidative stress, inflammatory mediators, apoptosis, nutritional disorders, and dysregulation of intestinal microecology [41]. Oxidative stress, in particular, involves the excessive production of reactive substances within the body under various environmental stresses or adverse conditions. This overproduction disrupts the balance between the body’s oxidative and antioxidant systems, resulting in tissue damage [42]. Oxidative stress induces several detrimental outcomes, including heightened intestinal mucosal permeability, intestinal damage, and susceptibility to intestinal infections. [43]. GSH and MDA could assess the level of oxidative stress in small intestinal tissues, and indirectly assess the degree of intestinal barrier damage [44]. Consequently, we assessed the concentrations of GSH and MDA in the small intestines of mice to examine the impact of indole derivatives on oxidative stress and the protective effects on the intestinal barrier in mice acutely infected with *T. gondii*, as illustrated in Figure 6a,b. Infection with *T. gondii* significantly reduced the GSH levels in the intestinal tissues of the mice compared to healthy controls. With the exception of derivatives **A1** and **A3**, indole, **A5**, **A8**, **A9**, and Spy did not mitigate the decrease in GSH levels in the small intestines of the infected mice. Notably, the GSH level in mice treated with derivative **A3** was significantly higher than that observed with the positive control drug, Spy, which also effectively counteracted the reduction in GSH levels in the small intestines of the infected mice. Conversely, *T. gondii* infection led to a significant increase in MDA levels in the intestinal tissues. Derivatives **A1**, **A3**, **A5**, **A8**, **A9**, and Spy demonstrated effective inhibition of the MDA increase induced by the infection. Notably, the efficacy of **A1** surpassed that of the positive control drug, Spy.

*T. gondii* typically results in oxidative stress and organ damage, which is attributed to an imbalance in the host immune system. This damage is particularly pronounced in the small intestine [45]. It possesses the capacity to scavenge hydroxyl free radicals and is depleted during instances of severe oxidative stress [46]. MDA was a product of oxidative stress and could be used as a marker to detect oxidative stress [47]. Indole derivative **A3** demonstrated a significant capacity to mitigate the reduction in GSH and the elevation of MDA levels induced by *T. gondii* infection. This suggests that the oxidative stress associated with *T. gondii* infection may be alleviated following the administration of **A3**.

To further investigate the protective effects of indole derivatives on the intestines of mice infected with *T. gondii*, we evaluated the levels of nitric oxide (NO) in the small intestine and diamine oxidase (DAO) in serum, as illustrated in Figure 6c,d. Derivatives **A1**, **A3**, **A5**, **A8**, and **A9** demonstrated the ability to mitigate the elevated levels of NO in the small intestine and DAO in the serum of infected mice. However, the reduction in NO levels following the administration of **A3** was less pronounced compared to the other derivatives, while its effect on DAO levels was more significant. The overproduction of NO, induced by endotoxins or endogenous inflammatory mediators, is associated with compromised mucosal barrier function, which can lead to increased intestinal permeability and, subsequently, intestinal barrier failure [48]. Conversely, NO also plays a role in enhancing the immune response against foreign pathogens, thereby aiding in the eradication of pathogenic microorganisms. Therefore, the degree of simulating proper inflammatory response was important [49,50]. Indole derivative **A3** showed ability to maintain the NO level and alleviate intestine damage in the small intestine of mice with *T. gondii* infection. This situation was odd, especially in preclinical research.

#### 2.3.5. Study on the Survival Rate in Mice with *T. gondii* Infection of Indole Derivatives

**Figure 7:**
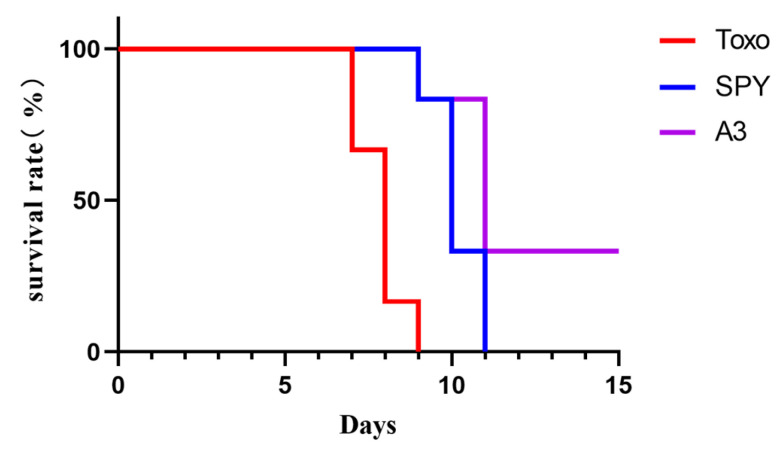
The effect of indole derivatives on survival rate of mice with *T. gondii* in vivo.

Due to the superior protective effect demonstrated by derivative **A3**, we further investigated its impact on the survival rate, as illustrated in Figure 7. During the acute phase of *T. gondii* infection, characterized by the rapid proliferation of tachyzoites, infected mice exhibited several symptoms, including piloerection, hypothermia, abdominal swelling, visible white discharge in the corners of the eyes, and a gradual decrease in appetite. By day seven, mice in the Toxo group ceased eating and drinking, ultimately leading to death. In contrast, administration of Spy extended the survival time of mice infected with *T. gondii*; however, their physiological condition did not show significant improvement. Notably, one-third of the infected mice survived. Although these surviving mice initially experienced a drop in body temperature, piloerection, and loss of appetite during the early observation period, these symptoms gradually resolved, and the mice eventually returned to a normal state.

#### 2.3.6. Study on the Mechanism of Indole Derivatives Against *T. gondii* Based on Molecular Docking

The inhibitory effect on NTPase leads to the death of tachyzoites, and the effect on TgCDPK1 could decrease the invasion capability, especially as TgCDPK1 is only present in protozoans and plants. Therefore, NTPase and TgCDPK1 could be considered as a potential target for drug synthesis against *T. gondii*. In the present study, indole derivatives were molecularly docked with NTPase-II and TgCDPK1 to investigate the mode of action of their anti-*T. gondii* activity. In general, binding energies less than −4.25 kcal/mol were thought as active for ligand–receptor binding, less than −5.0 kcal/mol for good binding activity, and less than −7.0 kcal/mol for strong binding activity. As shown in Table 3, Indole derivatives **A3**, **A5**, **A8**, and **A9** all showed strong binding activity to the NTPaseII enzyme.

The aldehyde groups in indole derivatives **A3**, **A5**, **A8**, and **A9** have hydrogen-bonding interactions with the arginine Arg)-300 residue, whereas the aldehyde group in indole derivative **A1** not only interacts with Arg-300 but also forms a carbon–hydrogen bond with serine (Ser)-468 residue. No valence bonding interaction with NTPase-II was observed for the raw materials indole and the indole derivative **A7**, which did not introduce an aldehyde group. It is therefore inferred that the formation of a hydrogen bond between the aldehyde group and Arg-300 is essential for the binding of indole derivatives to the NTPase-II enzyme.

**Figure 8:**
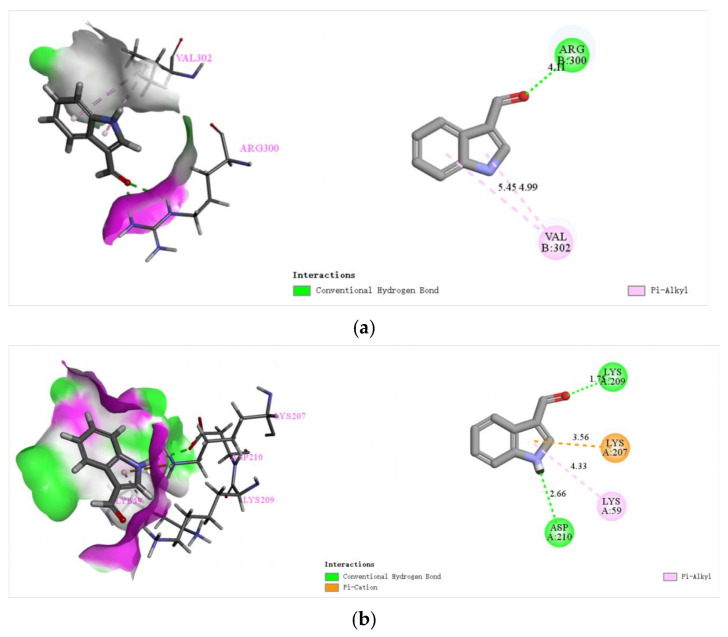
The mechanism of **A3** on anti-*T. gondii* in silica. (**a**) The molecular docking simulation of **A3** on NTPase; (**b**) the molecular docking simulation of **A3** on TgCDPK1.

**A3** showed better anti-*T. gondii* infection effects, but the mechanism was not clear, so we attempted to simulate the bond effect between **A3** and TgCDPK1. In the structure of the **A3** derivative, not only does the nitrogen–hydrogen bond on the indole ring interact with aspartic acid (Asp-210), but the aldehyde group also forms a hydrogen bond with lysine (Lys-209), which may be an important reason for the stable binding of **A3** to TgCDPK1 and an important factor for the good biological activity of **A3**, as shown in Figure 8. These two phenomena indicate that there exists a good transport mechanism of indole derivatives during the replication process of *T. gondii* in host cells, in which the indole of the peptidomimetic structure and the aldehyde group, which can easily form hydrated aldehydes in the microenvironment, are both suitable acceptors of hydrogen bonding, and these hydrogen-bonding interactions may be the key for the indole derivatives to have good anti-*T. gondii* activity and intestinal barrier protection, which provides a strong scientific basis for the development of anti-*T. gondii* chemicals. This provides a direction for the oriented modification of anti-*T. gondii* medicines and a strong scientific basis for the development of anti-*T. gondii* drugs.

### 2.4. SAR (Structure–Acitivity Relationship) Analysis of Indole Aldehyde Derivatives on Anti-T. gondii Activity

The results of the in vivo study indicated that compound **A3** exhibited superior anti-*T. gondii* activity compared to the control drug, Spy. Specifically, **A3** achieved a tachyzoite inhibition rate of 33.3%, which surpassed Spy’s rate of 31.3%. Additionally, oral **A3** administration mitigated the increase in serum diamine oxidase (DAO) and the levels of malondialdehyde (MDA) and nitric oxide (NO) in small intestinal tissues caused by acute *T. gondii* infection. It also elevated glutathione (GSH) levels in the small intestinal tissues. The gastrointestinal transport test further demonstrated that the indole derivative **A3** could alleviate the overall functional damage to the small intestine induced by *T. gondii* infection. Histopathological analysis and scoring also confirmed that **A3** provided a protective effect against intestinal barrier damage. The protective properties of **A3** may be attributed to the presence of an aldehyde group at the third position of the indole ring. This structural feature is shared with other biologically active indole compounds found in animals and plants, such as tryptophan and the plant growth hormone indole-3-acetic acid, which also have substituents at the third position. Notably, classical antitumor drugs such as ositinib and anilotinib feature modifications at this position [51,52,53]. This suggests that the modification of the third position of the indole is a common strategy in drug design, underscoring its significance in enhancing biological activity. Aldehydes easily form hydrogen-bonded receptors in the microenvironment, and the splicing of aldehyde groups at position 3 enhances the aqueous solubility of indole compounds, which promotes their uptake in vivo, and results in the better anti-*T. gondii* activity of indole derivative **A3**, both in vivo and in vitro.

The selectivity index for anti-*T. gondii* activity of compound **A4** (SI = 1.0) was found to be lower than that of the control drug Spy (SI = 1.1). This reduced efficacy may be attributed to the substitution of a benzene ring with an aniline group, potentially destabilizing the molecular structure and thereby diminishing biological activity. Comparative analysis of in vitro data for compounds **A3**, **A4**, **A5**, and **A7** revealed a hierarchy in selectivity indices: **A3** > **A5** > **A4** = **A7**. Notably, compound **A7**, lacking an aldehyde group, exhibited a lower selectivity index (SI = 1.0) compared to **A5**, which includes an aldehyde group at the third position on the indole ring. This suggests that the aldehyde group contributes to mitigating intestinal barrier damage induced by *T. gondii* in mice. Compound **A3**, with the highest selectivity index (SI = 1.3), incorporates an aldehyde group at the same position, further supporting this observation. Further analysis, integrating in vivo data on glutathione (GSH), malondialdehyde (MDA), nitric oxide (NO), diamine oxidase (DAO), and histopathological sections, indicated that compounds **A3** and **A5** ameliorate intestinal barrier damage in acute *T. gondii* infections. This protective effect is likely mediated through the modulation of oxidative stress and inflammation, which mitigates the intestinal damage. Among these, indole derivative **A3** demonstrated superior anti-T. gondii activity compared to **A5**. This enhanced activity may be due to the presence of a chlorine atom at the second position in **A5**, which increases the compound’s oxidizing properties. In vivo studies of compounds **A8** and **A9** showed that **A8** slightly outperformed **A9** in reducing MDA and NO levels in small intestinal tissues. However, the DAO levels in the serum of the **A9** group did not significantly differ from those in the Toxo group. These findings suggest that the introduction of a benzene ring at the second position might reduce the compound’s bioactivity, even under conditions where an aldehyde group is introduced at the first position and a methyl group at both of the third positions. Consistent with the results of molecular docking, the aldehyde group in the structure of compound **A3** formed hydrogen bonds not only with the Arg-300 residue of NTPaseII, but also with the Lys-209 residue of TgCDPK1. Additionally, the nitrogen–hydrogen bonds on the indole ring engaged in hydrogen-bonding interactions with the Asp-210 residue. These interactions likely contribute to the enhanced anti-*T. gondii* activity and intestinal barrier protection effects observed with this compound.

## 3. Materials and Methods

### 3.1. Chemistry

**Figure 9:**
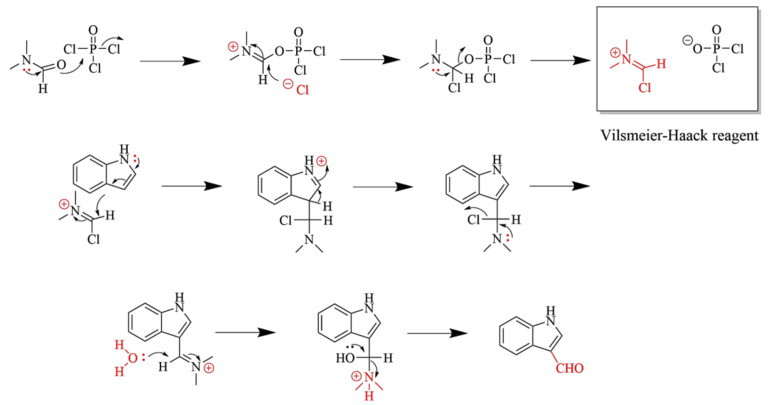
The mechanism of Vilsmeier–Hacck reaction mechanism.

High-grade chemical reagents and solvents were obtained from commercial vendors (Aladin, Shanghai, China; Kemiou, Tianjin, China; Macklin, Shanghai, China; Adamas-beta, Shanghai, China; Sigma Aldrich, St. Louis, MO, USA; Energy Chemical, Shanghai, China; Thermo Fisher Scientific, Waltham, MA, USA; Bidepharm, Shanghai, China) and were used without additional purification. Thin-layer chromatography (TLC) was performed on precoated TLC plates (SiliaPlate TLC, thickness 200 µm, GF-254, Qingdao Haiyang Chemical Co., Ltd., Qingdao, Shandong, China) and visualized under a UV lamp (λ = 254 and 365 nm). Chromatographic preparative separations were accomplished in glass columns filled with silica gel (SiliaFlash, particle size 44–65 µm, Qingdao Haiyang Chemical Co., Ltd., Qingdao, Shandong, China). Nuclear magnetic resonance (NMR) spectra (1D: ^1^H and ^13^C) were recorded on an AV-NEO 300 MHz spectrometer (Bruker, Fällanden, Switzerland). LC-MS-MS measurements were recorded on a 1200RR LC-6410B (Agilent, Denver, CA, USA) operating in electrospray mode (ESI) with positive ionization. The Vilsmeier–Hacck reaction mechanism is shown in Figure 9.

#### 3.1.1. Synthesis of Derivative **A1**

A total of 500 μL of *N,N*-dimethylformamide was placed in a round-bottomed flask under stirring and an ice bath for 1 h. Then, 90 μL of phosphorus triclosan was added to *N,N*-dimethylformamide and the flask was cooled in an ice bath for 1 h. A total of 100 mg of 3-indoleacetonitrile was weighed and dissolved in 500 μL of *N,N*-dimethylformamide in an ice bath for 1 h. The flask was heated and stirred at 60 °C for 1 h. An equal volume of crushed ice was added to the resulting clarified aqueous solution and refluxed for 10 min until the solution was neutral. After adding an equal volume of crushed ice, sodium bicarbonate solution was added to the resulting clarified aqueous solution until the solution was neutral, then it was refluxed for 10 min, cooled to room temperature and placed in a partition funnel, extracted with dichloromethane, dried in a conical flask containing anhydrous magnesium sulfate, filtered, spun-dried, and the product was isolated and purified by silica gel column chromatography to obtain the derivative **A1**.

#### 3.1.2. Synthesis of Derivative **A2**

A total of 500 μL of *N,N*-dimethylformamide was placed in a round-bottomed flask under stirring and an ice bath for 1 h. Then, 30 μL of phosphorus triclosan was added to *N,N*-dimethylformamide and the flask was cooled in an ice bath for 1 h. A total of 40 mg of 2-(3-Indole)-ethylamine was weighed and dissolved in 500 μL of *N,N*-dimethylformamide in an ice bath for 1 h. The flask was heated and stirred at 60 °C for 24 h. An equal volume of crushed ice was added to the resulting clarified aqueous solution and refluxed for 10 min until the solution was neutral. After adding an equal volume of crushed ice, sodium bicarbonate solution was added to the resulting clarified aqueous solution until the solution was neutral, then it was refluxed for 10 min, cooled to room temperature and placed in a partition funnel, extracted with dichloromethane, dried in a conical flask containing anhydrous magnesium sulfate, filtered, spun-dried, and the product was isolated and purified by silica gel column chromatography to obtain the derivative **A2**.

#### 3.1.3. Synthesis of Derivative **A3**

A total of 500 μL of *N,N*-dimethylformamide was placed in a round-bottomed flask under stirring and an ice bath for 1 h. Then, 60 μL of phosphorus triclosan was added to *N,N*-dimethylformamide and the flask was cooled in an ice bath for 1 h. A total of 50 mg of indole was weighed and dissolved in 500 μL of *N,N*-dimethylformamide in an ice bath for 1 h. The flask was heated and stirred at 35 °C for 1 h. An equal volume of crushed ice was added to the resulting clarified aqueous solution and refluxed for 10 min until the solution was neutral. After adding an equal volume of crushed ice, 0.5 mL of 40% sodium hydroxide solution was added to the resulting clarified aqueous solution until the solution was neutral, then it was refluxed for 10 min, cooled to −4 °C overnight, and the precipitate was filtered and washed twice with distilled water to obtain the derivative **A3**.

#### 3.1.4. Synthesis of Derivative **A4**

A total of 500 μL of *N,N*-dimethylformamide was placed in a round-bottomed flask under stirring and an ice bath for 1 h. Then, 30 μL of phosphorus triclosan was added to *N,N*-dimethylformamide and the flask was cooled in an ice bath for 1 h. A total of 40 mg of 5-bromo-7-dideazapurine was weighed and dissolved in 500 μL of *N,N*-dimethylformamide in an ice bath for 1 h. The flask was heated and stirred at 60 °C for 24 h. An equal volume of crushed ice was added to the resulting clarified aqueous solution and refluxed for 10 min until the solution was neutral. After adding an equal volume of crushed ice, sodium bicarbonate solution was added to the resulting clarified aqueous solution until the solution was neutral, then it was refluxed for 10 min, cooled to room temperature, and placed in a partition funnel, extracted with dichloromethane, dried in a conical flask containing anhydrous magnesium sulfate, filtered, spun-dried, and the product was isolated and purified by silica gel column chromatography to obtain the derivative **A4**.

#### 3.1.5. Synthesis of Derivatives **A5** and **A7**

A total of 500 μL of *N,N*-dimethylformamide was placed in a round-bottomed flask under stirring and an ice bath for 1 h. Then, 20 μL of phosphorus triclosan was added to *N,N*-dimethylformamide and the flask was cooled in an ice bath for 1 h. A total of 20 mg of oxindole was weighed and dissolved in 500 μL of *N,N*-dimethylformamide in an ice bath for 1 h. The flask was heated and stirred at 60 °C for 24 h. An equal volume of crushed ice was added to the resulting clarified aqueous solution and refluxed for 10 min until the solution was neutral. After adding an equal volume of crushed ice, sodium bicarbonate solution was added to the resulting clarified aqueous solution until the solution was neutral, then it was refluxed for 10 min, cooled to room temperature, and placed in a partition funnel, extracted with dichloromethane, dried in a conical flask containing anhydrous magnesium sulfate, filtered, spun-dried, and the product was isolated and purified by silica gel column chromatography to obtain the derivatives **A5** and **A7**.

#### 3.1.6. Synthesis of Derivative **A6**

A total of 500 μL of *N,N*-dimethylformamide was placed in a round-bottomed flask under stirring and an ice bath for 1 h. Then, 30 μL of phosphorus triclosan was added to *N,N*-dimethylformamide and the flask was cooled in an ice bath for 1 h. A total of 40 mg of oxindole was weighed and dissolved in 500 μL of *N,N*-dimethylformamide in an ice bath for 1 h. The flask was heated and stirred at 60 °C for 24 h. An equal volume of crushed ice was added to the resulting clarified aqueous solution and refluxed for 10 min until the solution was neutral. After adding an equal volume of crushed ice, sodium bicarbonate solution was added to the resulting clarified aqueous solution until the solution was neutral, then it was refluxed for 10 min, cooled to room temperature, and placed in a partition funnel, extracted with dichloromethane, dried in a conical flask containing anhydrous magnesium sulfate, filtered, spun-dried, and the product was isolated and purified by silica gel column chromatography to obtain the derivative **A6**.

#### 3.1.7. Synthesis of Derivative **A8**

A total of 500 μL of *N,N*-dimethylformamide was placed in a round-bottomed flask under stirring and an ice bath for 1 h. Then, 250 μL of phosphorus triclosan was added to *N,N*-dimethylformamide and the flask was cooled in an ice bath for 1 h. A total of 131.2 mg of 1-methylindole was weighed and dissolved in 500 μL of *N,N*-dimethylformamide, and the flask was heated and stirred at 60 °C for 1 h. A volume of crushed ice was added to the resulting clarified aqueous solution until the solution was neutral, then it was refluxed for 10 min, and cooled to room temperature. Then, sodium bicarbonate solution was added to the resulting clarified aqueous solution until the solution was neutral, then it was refluxed for 10 min, cooled to room temperature, and placed in a separatory funnel, extracted with dichloromethane, dried in a conical flask containing an-hydrous magnesium sulfate, filtered, spun-dried, and the product was isolated and purified by silica gel column chromatography to obtain the derivative **A8**.

#### 3.1.8. Synthesis of Derivative **A9**

A total of 1 mL of *N,N*-dimethylformamide was placed in a round-bottomed flask under stirring and an ice bath for 1 h. Then, 73.6 μL of phosphorus triclosan was added to *N,N*-dimethylformamide and the flask was cooled in an ice bath for 1 h. A total of 100 mg of 1-Methyl-2-phenyl-1*H*-indole was weighed and dissolved in 500 μL of *N,N*-dimethylformamide, and the flask was heated and stirred at 25 °C for 12 h. A volume of crushed ice was added to the resulting clarified aqueous solution until the solution was neutral, then it was refluxed for 10 min, and cooled to room temperature. Then, sodium bicarbonate solution was added to the resulting clarified aqueous solution until the solution was neutral, then it was refluxed for 10 min, cooled to room temperature, and placed in a separatory funnel, extracted with dichloromethane, dried in a conical flask containing an-hydrous magnesium sulfate, filtered, spun-dried, and the product was isolated and purified by silica gel column chromatography to obtain the derivative **A9**.

### 3.2. Biological Activity

Dulbecco’s Modified Eagle Medium (DMEM, HyClone^®^, Waltham, MA, USA), Trypsin-EDTA (Solarbio, Beijing, China), Penicillin-Streptomycin solution (Solarbio, Beijing, China) and fetal bovine serum (FBS, Gibco^®^, Grand Island, NY, USA) were used for cell culture. Spiramycin (Spy) was purchased from Aladdin Industrial Inc. (Shanghai, China). The compound was dissolved in dimethylsulfoxide (DMSO, Solarbio, Beijing, China) and diluted with DMEM to different concentrations, with the final DMSO concentration at less than 1% (*v*/*v*). All other chemicals were of reagent grade and purchased from Aladdin Industrial Inc. (Shanghai, China). Other high-grade chemical reagents and solvents were obtained from commercial vendors (Aladin, Shanghai, China).

#### 3.2.1. Cells, Parasites and Animals

IEC-6 cells were cultured in DMEM, supplemented with 0.01% Penicillin-Streptomycin and 10% heat-inactivated FBS and maintained at 37 °C and 5% CO_2_. Cells were purchased from American Type Culture Collection (ATCC, Manassas, VA, USA). Tachyzoites were from the virulent RH strain of *T. gondii* maintained by serial intraperitoneal passage in KM female mice, which were purchased from Experiment Center, Yanbian University (Yanji, Jilin, China). All experimental procedures were conducted in accordance with institutional guidelines for the care and use of laboratory animals at Yianbian University and conformed to the National Institutes of Health Guide or Care and Use of Laboratory Animals (license number SCXK 2017-0003). All mice were kept in a central animal care facility with free access to water and rodent food during the experiment.

#### 3.2.2. Anti-*T. gondii* Activity and Cytotoxicity of Indole Derivatives In Vitro

Anti-*T. gondii* activity and cytotoxicity of indole derivatives in vitro were evaluated by MTT assay (Solarbio, Beijing, China) using a slightly modified version of the procedure of Shang et al. [54]. IEC-6 cells were seeded onto the 96-well plates (2 × 10^3^/well) for 24 h to obtain the full monolayer, and then the host cells were infected with *T. gondii* (parasite: cell = 3:1) in complete medium. After 24 h, cells were washed to remove any extra parasites and then incubated with different concentrations (20, 200, 500 and 1000 μm) of total polysaccharides and saponins. Spy served as the positive control and DMSO as the negative control. After 24 h of treatment, MTT solution was added directly to the culture wells and then incubated for 4 h at 37 °C. The optical absorbance was measured at a wavelength of 548 nm using a microplate reader (SpectraMax M2, Molecular Devices, Sunnyvale, CA, USA). Giemsa staining was used to observe the changes in cell morphology and the proliferation of *T. gondii*-infected cells. IEC-6 (1 × 10^5^ cells/well) was inoculated into 24-well plates containing round coverslip, and after the cells were attached to the wall, *T. gondii* (3 × 10^5^ cells/well) was added to infect the cells for 3 h. After the culture medium was discarded, PBS was added to remove the uninvaded tachyzoites. The drug was administered for 12 h according to the IC_50_ value of the cells obtained by MTT, the positive control drug group was Spy, and the negative control group was given culture medium only. After discarding the supernatant, the cells were washed with PBS and completely aspirated, and then fixed by adding 0.5 mL of methanol for 3 min. Giemsa staining solution was added and shaken slightly to make the staining uniform. Coverslips were rinsed with distilled water to replace the staining solution, and finally, the slices were sealed with neutral gum and air-dried. After drying, the slices were fixed on slides with neutral resin, and the intracellular infection and proliferation of *T. gondii* after treatment with indole derivatives were observed under the microscope (CKX53, OLYMPUS, Tokyo, Japan) and recorded by a digital camera with a microscope (EP50, OLYMPUS, Tokyo, Japan),.

#### 3.2.3. Anti-*T. gondii* Activity of Indole Derivatives In Vivo

For the in vivo study, KM female mice were divided into groups consisting of six animals each and then intraperitoneally injected with 2 × 10^3^ tachyzoites. After 4 h infection, mice were orally treated or not with different compounds: 0.2 mL normal saline (normal group and negative control group), 100 mg/kg of **A1**, **A3**, **A5**, **A8**, **A9**, Spy, respectively, once a day for 6 days. On the last day, heart blood samples were collected after anesthesia to separate the serum, and after mice were sacrificed by cervical dislocation, mice were dissected to obtain organs and tissues. The tachyzoites were harvested from mice peritoneal cavities to determine the level of tachyzoite proliferation [55]. The peritoneal fluid was collected twice; the first time, it was centrifuged at 500 rpm for 10 min, then the supernatant was centrifuged at 2500 rpm for 20 min, discarded, and the worm precipitate was collected at the bottom, and mixed with an appropriate amount of physiological saline. A total of 10 μL of *T. gondii* suspension was mixed with 10 μL of 4% Trypan blue staining solution in a 1.5 mL centrifuge tube. A total of 10 μL of the solution was placed on a cell counting plate and the number of tachyzoites was recorded under a light microscope to calculate the inhibition rate of *T. gondii*.

#### 3.2.4. Intestine Barrier Protective Effects of Mice with *T. gondii* Infection in Apparent Change

Mouse food intake and body weight were recorded daily for six days during the experiment and were used to record growth performance data. At the end of the experiment, body weight gain, food intake and food utilization of mice were calculated as Equation (1).

(1) $$food\;utilization\;rate(\%)=\frac{weight\;increment}{food\;intake\;weight}\times 100\%$$

A solution containing 3% carmine dye and 0.5% carboxymethyl cellulose was prepared. On the fifth day of *T. gondii* infection, each mouse was transferred to a separate cage, with the bottom of the cage completely covered with white paper to facilitate the detection of cochineal in the feces after red feces were eliminated. After the mice had adapted to the environment, each mouse was filled by gavage with 0.25 mL of cochineal solution and was observed at 0.5 h intervals, and records were kept until all mice excreted the cochineal. During the experimental period, the mice were free to move around, and eat and drink freely without any external interference, as described [56].

The histology research includes apparent pathological change score, H&E staining and histopathological scores of tissue [57], as shown in Table 4 and Table 5. In detail, the obtained small intestine tissues were fixed in formalin solution and embedded in paraffin wax after gradual dehydration with different concentrations of ethanol. The paraffin fast was sectioned to a thickness of 5 μm, rehydrated and stained with hematoxylin and eosin, respectively, and finally permeabilized with xylene and sealed with neutral gum. A light microscope was used to observe and collect pictures, and the degree of damage to the small intestinal tissues was assessed according to Chiu’s six-grade scale.

#### 3.2.5. Intestine Barrier Protective Effects of Mice with *T. gondii* Infection in Biochemical Parameters

The GSH was measured according to the method in [58]. The intestine homogenate was mixed with half volume trichloroacetic acid (20%, *w*/*v*) and centrifuged at 4000 rpm for 10 min. Then, phosphate buffer (phosphate 0.3 mol/L, pH 7.5) and 5,5-dithio-bis-(2-nitrobenzoic acid) (0.04%, *w*/*v*) were added to the separated supernatant and mixed thoroughly. After 5 min at room temperature, the absorbance was measured at 412 nm.

MDA was measured by the standard method [59] with minor modifications. The small intestine homogenate supernatant was mixed with thiobarbituric acid (0.5%, *w*/*v*) and heated in boiling water bath for 1 h, then cooled quickly and centrifuged at 6000 rpm for 10 min; the absorbance of pink colored supernatant was measured at 532 nm. Tetraethoxypropane replaced the intestine homogenate in the standard sample.

The Greiss method was used to determine the NO content in small intestine tissues and cells. The above small intestine tissue homogenate or IEC-6 cell supernatant was centrifuged at 4 °C and 1500 rpm for 15 min, and the absorbance was measured at 540 nm by adding equal amounts of Griess A and B reagents to the supernatant. The standard used in the NO standard curve was sodium nitrite, and the NO content in the samples was calculated by substituting the absorbance values of the samples into the regression equation of the standard curve.

The DAO evaluation experimental method of Xin Y et al. was adopted [60], in which mouse heart blood was taken from a 1.5 mL centrifuge tube and centrifuged at 4 °C and 3000× *g* for 10 min, and the upper layer of serum was taken for measurement. Another 1.5 mL tube was taken and 600 μL of phosphate buffer, 20 μL of horseradish peroxidase, 20 μL of dianisidine dihydrochloride hydrochloride, and 20 μL of 1,5-pentanediamine hydrochloride were added. After that, 100 μL of serum samples to be tested was added, mixed well and heated at 37 °C for 18 h. The absorbance was measured at 436 nm. The absorbance was measured at 436 nm. A total of 100 μL of distilled water was used in the blank tube instead of serum, and the standard used in the standard curve was diamine oxidase. The absorbance value of the experimental samples was substituted into the regression equation of the standard curve to calculate the content of DAO in the serum samples.

#### 3.2.6. The Investigation of Anti-*T. gondii* Mechanism of Indole Derivatives by Molecular Docking

Molecular docking analysis can provide important insights into ligand–enzyme binding and conformation, and in order to explore the mechanism of action of indole derivatives for the treatment of toxoplasmosis, in this study, we used the NTPase-II enzyme and the calcium-dependent protein kinase 1 TgCDPK1) of *T. gondii* as receptor targets, and molecular docking was performed in the CDOCKER mode using the Discovery Studio 2017 software (DNASTAR, Madison, WI, USA). The 3D structures of NTPase-II enzyme and TgCDPK1 were first searched in the RCSB PDB protein database PDB ID:4KH5, 3N51). The receptor was imported into Discovery Studio 2017 Client and preprocessed. Other ligand molecules, such as water molecules, were removed. We clicked on Tools—Macromolecules—Prepare Protein—Clean Protein to carry out the steps, such as hydrogenation for the protein. The indole analog was opened in Chem Draw and saved as a .dsv file. The ligands were imported into Discovery Studio 2017 Client and preprocessed; Tools—Receptor–Ligand Interactions—Dock Ligands—Prepare Ligands was used to obtain indole analog-related files. Subsequently, the Binding Site was defined using the following sequence: Tools—Receptor–Ligands Interaction—Define and Edit Binding Site—Select Ligands—From Current Selection—Delete Ligands. Finally, the molecular docking simulation was performed using the following sequence: Tools—Receptor–Ligand Interactions—Dock Ligands—Dock Optimization—Dock ligand. This was carried out to complete the molecular docking, and the docking result with the highest ligand–receptor binding energy was selected as a reference.

#### 3.2.7. Statistical Analysis

All data were expressed as mean ± standard deviation (S.D.) in triplicate. Statistical analyses and graphs were performed using SPSS 16.0 software (SPSS Inc., Chicago, IL, USA) and GraphPad Prism 5.0 (GraphPad Software Inc., San Diego, CA, USA). The method of Kaplan and Meier was used to compare the survival rates of the studied groups. A value of *p* < 0.05 was considered statistically significant.

## 4. Conclusions

Although the specific mechanism was not investigated clearly, our result shows the effects on anti-*T. gondii* activity and small intestine protection of indole and derivatives **A1**, **A3**, **A5**, **A8**, **A9**. This interaction is likely the basis for the derivatives’ potent anti-*T. gondii* activity and their protective effects on the intestinal barrier. These findings not only offer a new therapeutic avenue for treating intestinal barrier damage caused by *T. gondii* infection but also contribute to the advancement of treatments for toxoplasmosis. After the investigation of the anti-*T. gondii* mechanism, the further development of **A3** as a target toxoplasmosis drug is feasible.

## Figures and Tables

**Table 1:** In vitro anti-*T. gondii* activity of indole derivatives by MTT assay ^a^.

Compounds	IC50 ^b^ in IEC-6 Cells	IC50 ^c^ in *T. gondii*	Selective Index ^d^ (SI)
	(µM)	(µM)	
**Indole**	939.6 ± 5.4	921.2 ± 5.0	1.0
**A1**	416.7	342.5	1.2
**A2**	>1000	>1000	-
**A3**	869.2 ± 4.0	674.7 ± 8.3	1.3
**A4**	960.3 ± 2.5	995.7 ± 4.9	1.0
**A5**	602.5 ± 9.9	564.1 ± 6.9	1.1
**A6**	632.7 ± 9.8	741.9 ± 10.5	0.9
**A7**	283.7 ± 2.6	287.3 ± 6.1	1.0
**A8**	649.9 ± 7.9	571.2 ± 7.4	1.1
**A9**	408.4 ± 3.5	365.7 ± 8.8	1.1
**Spy**	246.4 ± 5.9	219.4 ± 6.0	1.1

^a^ Each value is expressed as the mean ± SD (*n* = 3). ^b^ IC50 in IEC-6 cells = concentration required to reduce IEC-6 cell growth by 50%. ^c^ IC50 in *T. gondii* = concentration required to reduce *T. gondii*-infected IEC-6 cell growth by 50%. ^d^ SI = selectivity index, a measure of efficacy, calculated by IC_50_ in IEC-6 cells/IC_50_ in *T. gondii*-infected IEC-6 cells. Values are expressed as mean ± S.D (*n =* 6).

**Table 2:** In vivo anti-*T. gondii* activity of indole derivatives by Trypan Blue assay ^a^.

Groups	Number of Tachyzoites (×10^6^)	Inhibition Rate (%) ^b^
**Toxo**	12.28 ± 0.39	-
**Indole**	10.33 ± 2.84	10.6
**A1**	8.17 ± 0.84 **	22.3
**A3**	8.22 ± 1.49 **	33.3
**A5**	9.56 ± 0.92 *	22.2
**A8**	8.92 ± 0.51 *	18.3
**A9**	8.83 ± 0.39 **	28.1
**Spy**	9.22 ± 1.73 *	31.3

^a^ Each value is expressed as the mean ± SD (*n* = 6). ^b^ Mice peritoneal *T. gondii* inhibition rate, calculated by (untreated group − treated group)/untreated group × 100%. * *p* < 0.05, ** *p* < 0.01, compared with Toxo group.

**Table 3:** The binding energy of tested compounds.

Groups	Binding Energy (kcal/mol)
**indole**	-
**A1**	−4.142
**A2**	−11.484
**A3**	−7.752
**A4**	−9.264
**A5**	−9.492
**A6**	−4.947
**A7**	-
**A8**	−10.612
**A9**	−7.285

**Table 4:** Small intestine histopathy score.

Score	Blood on the Outer Barrier	Arteriosclerosis	Digestive Fluid Content	Flatulence
1–2	few	few	few	few
3–4	many	many	many	many
5–6	magnanimous	magnanimous	magnanimous	magnanimous

**Table 5:** Chiu’s six-grade scale.

Score	Situation of Histopathology in H&E Staining
0	Normal mucosa and villi
1	Subepithelial appearance of Gruenhagen’s gap at the tip of intestinal villi with capillary dilatation
2	Marked enlargement of the subepithelial Gruenhagen gap
3	Cellular degeneration and necrosis of the epithelial layer of the intestinal mucosa, with apical detachment of a few villi
4	Epithelial cell layer degenerated, necrotic, detached, lamina propria exposed, some villi detached
5	Loss of villi, disintegration of lamina propria, hemorrhage or ulcer formation

## Data Availability

The raw data supporting the conclusions of this article will be made available by the authors on request.

## Supplementary Materials

The following supporting information can be downloaded at: https://www.mdpi.com/article/10.3390/molecules29215024/s1, Figures S1 and S2: Spectrum of indole; Figures S3 and S4: Spectrum of indole derivative **A1**; Figures S5–S8: Spectrum of indole derivative **A2**; Figures S9–S12: Spectrum of indole derivative **A3**; Figures S13 and S14: Spectrum of indole derivative **A4**; Figures S15 and S16: Spectrum of indole derivative **A5**; Figures S17–S19: Spectrum of indole derivative **A6**; Figures S20 and S21: Spectrum of indole derivative **A7**; Figures S22 and S23: Spectrum of indole derivative **A8**; Figures S24 and S25: Spectrum of indole derivative **A9**; Table S1: The water solubility, lipophilicity and drug-likeness prediction of indole derivatives; Figure S26: Pharmacokinetic prediction of indole; Figure S27: Pharmacokinetic prediction of **A1**; Figure S28: Pharmacokinetic prediction of **A2**; Figure S29: Pharmacokinetic prediction of **A3**; Figure S30: Pharmacokinetic prediction of **A4**; Figure S31: Pharmacokinetic prediction of **A5**; Figure S32: Pharmacokinetic prediction of **A6**; Figure S33: Pharmacokinetic prediction of **A7**; Figure S34: Pharmacokinetic prediction of **A8**; Figure S35: Pharmacokinetic prediction of **A9**.
