# Supplementary material for: The Intestinal Barrier Protective Effect of Indole Aldehyde Derivatives on Acute Toxoplasma gondii Infection
Source: Molecules. 2024 Oct 24;29(21):5024. doi: 10.3390/molecules29215024 (PMC11547840; doi:10.3390/molecules29215024)
Supplement: Supplementary file 1 [file molecules-29-05024-s001.zip › molecules-3265379-supplementary.pdf]

Supporting Information

The Intestinal Barrier Protective Effect of Indole Derivatives on Acute *Toxoplasma Gondii* Infection

Jieqiong Wang [1#], Weifeng Yan [1#], Xu Cheng [1], Yonggang Tong [1], Sihong Wang [2,*], and Chunmei Jin [1,*]

1 Key Laboratory of Natural Medicines of the Changbai Mountain, Ministry of Education, College of Pharmacy, Yanbian University, Yan'Ji 133002, China; Jieqiong990804@163.com (J.W.), 719246811@qq.com (W.Y.), 0000007999@ybu.edu.cn (X.C.), 2238418385@qq.com; (Y.T.).
2 Analysis and Inspection Center, Yanbian University, Yan'Ji 133002, China*
Correspondence: cmjin@ybu.edu.cn, shwang@ybu.edu.cn

1.Spectrum of indole 2

2.Spectrum of indole derivative A1...3

3.Spectrum of indole derivative A2...4

4.Spectrum of indole derivative A3...6

5.Spectrum of indole derivative A4...8

6.Spectrum of indole derivative A5...9

7.Spectrum of indole derivative A6...10

8.Spectrum of indole derivative A7...12

9.Spectrum of indole derivative A8...13

10.Spectrum of indole derivative A9...14

11.Pharmacokinetic prediction of compounds...15

1. *Spectrum of indole*

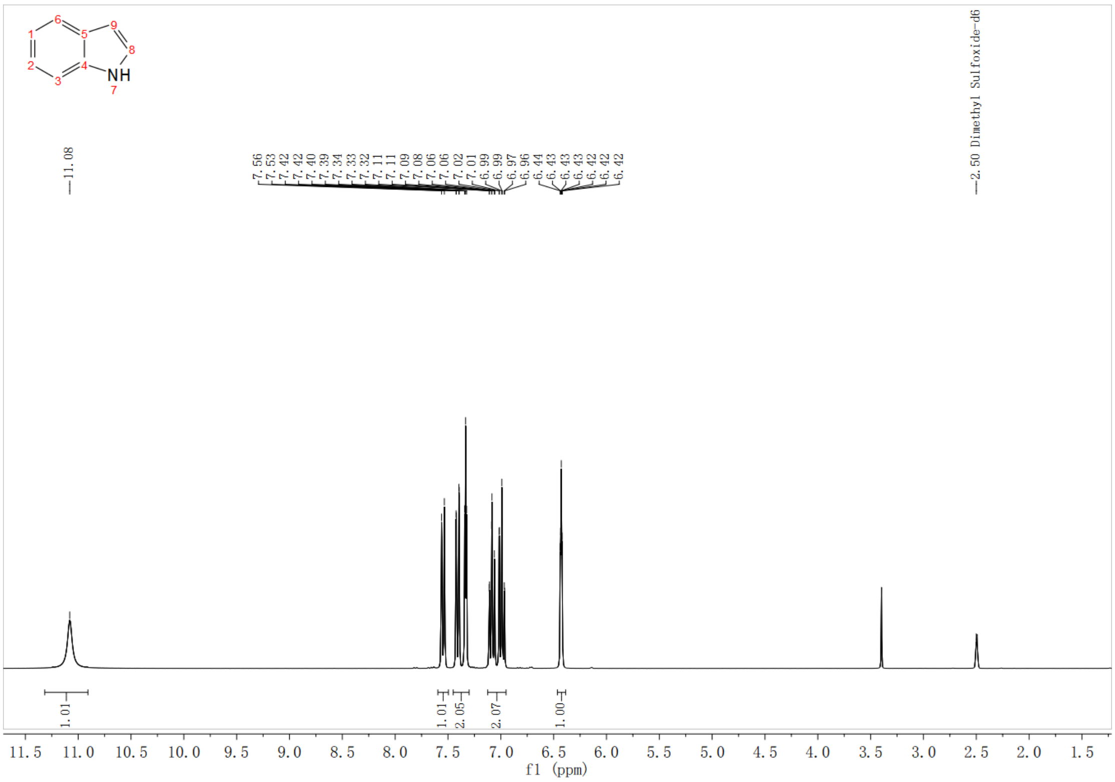

Figure S1. ¹H NMR spectrum of indole (CDCl₃, 300MHZ)

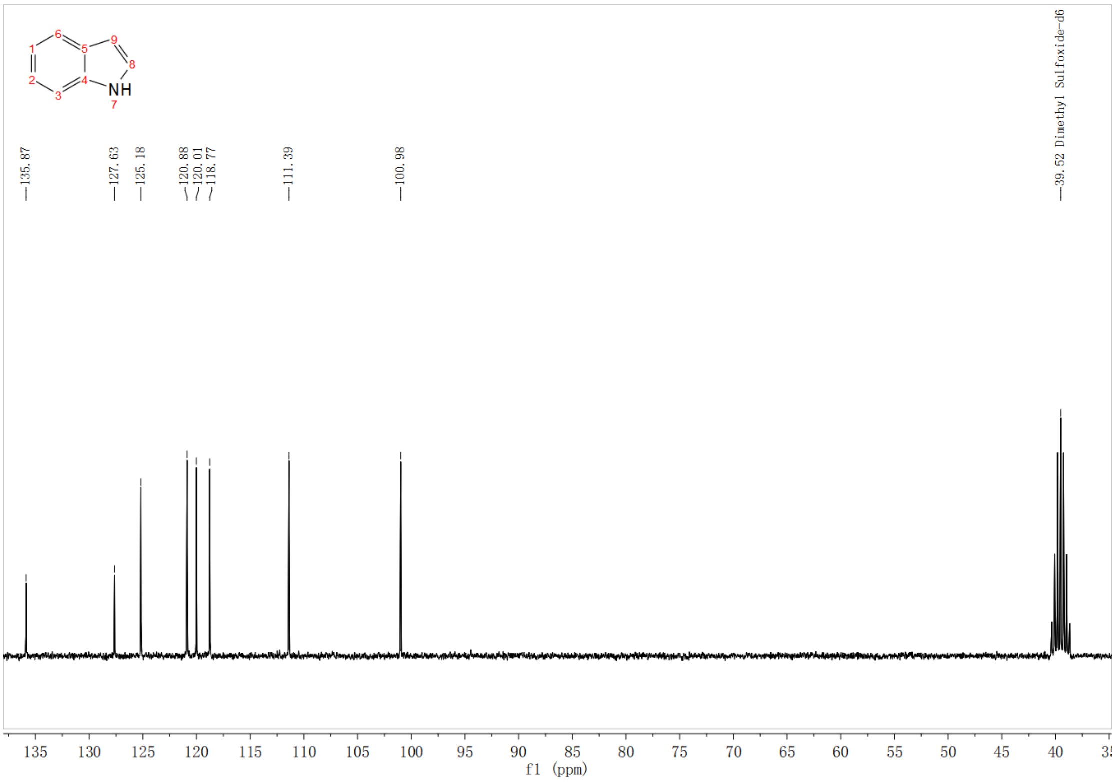

Figure S2. ¹³C NMR spectrum of indole (CDCl₃, 75MHZ)

2. *Spectrum of indole derivative A1*

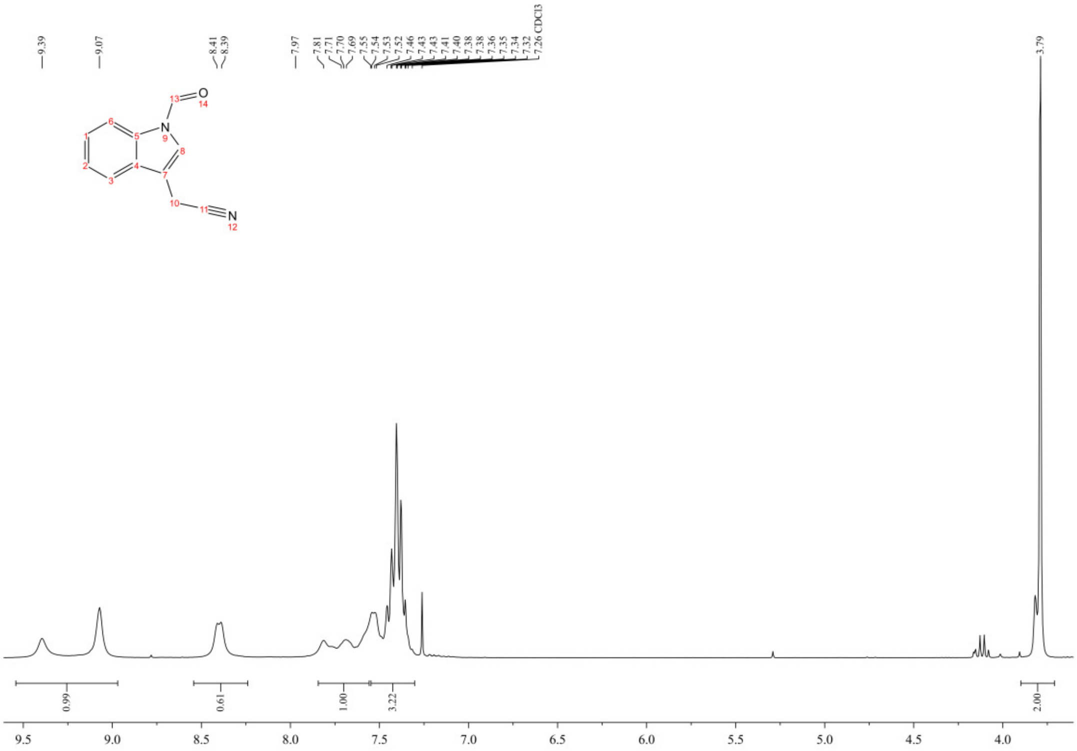

Figure S3. ^{1}H NMR spectrum of indole derivatives A1 ($CDCl_3$, 300MHZ)

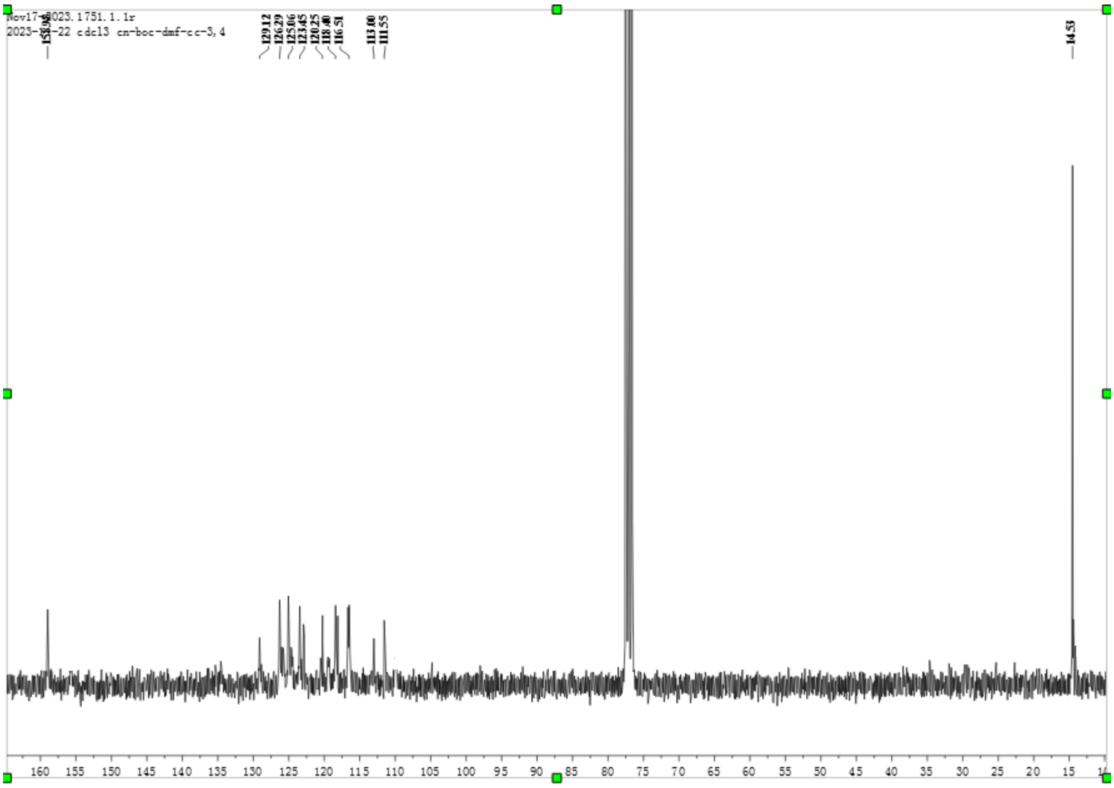

Figure S4. ^{13}C NMR spectrum of indole derivatives A1 ($CDCl_3$, 75MHZ)

3. Spectrum of indole derivative A2

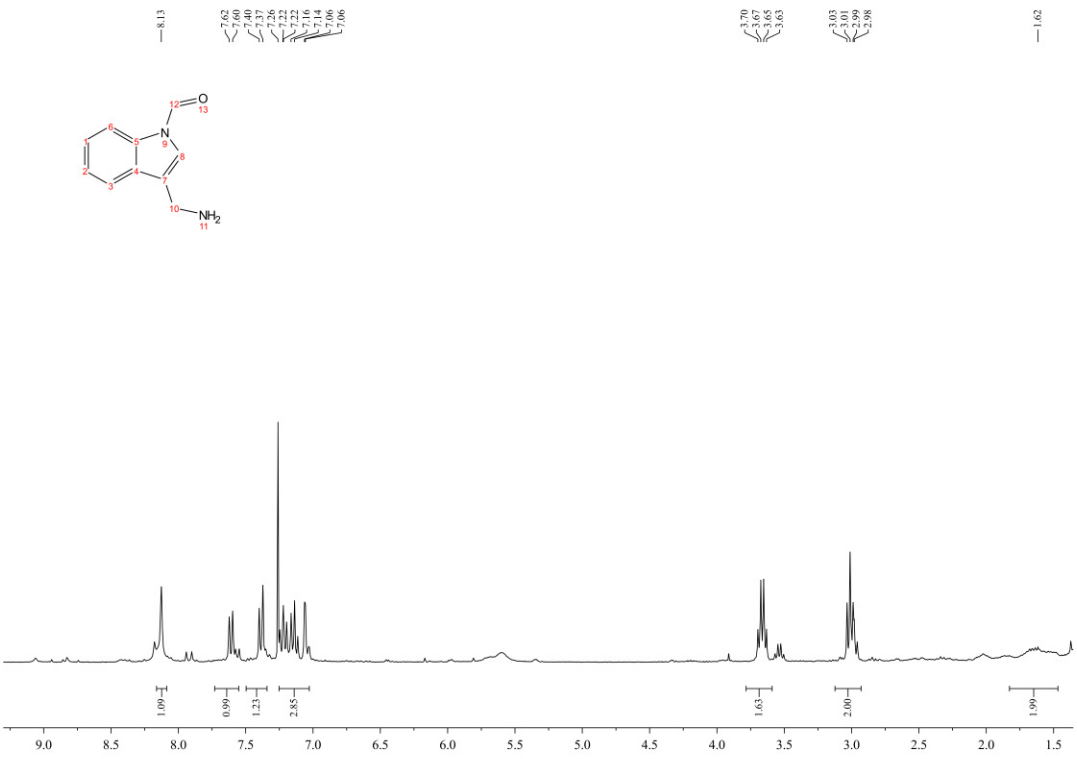

Figure S5. ^{1}H NMR spectrum of indole derivatives A2 ($CDCl_3$, 300MHZ)

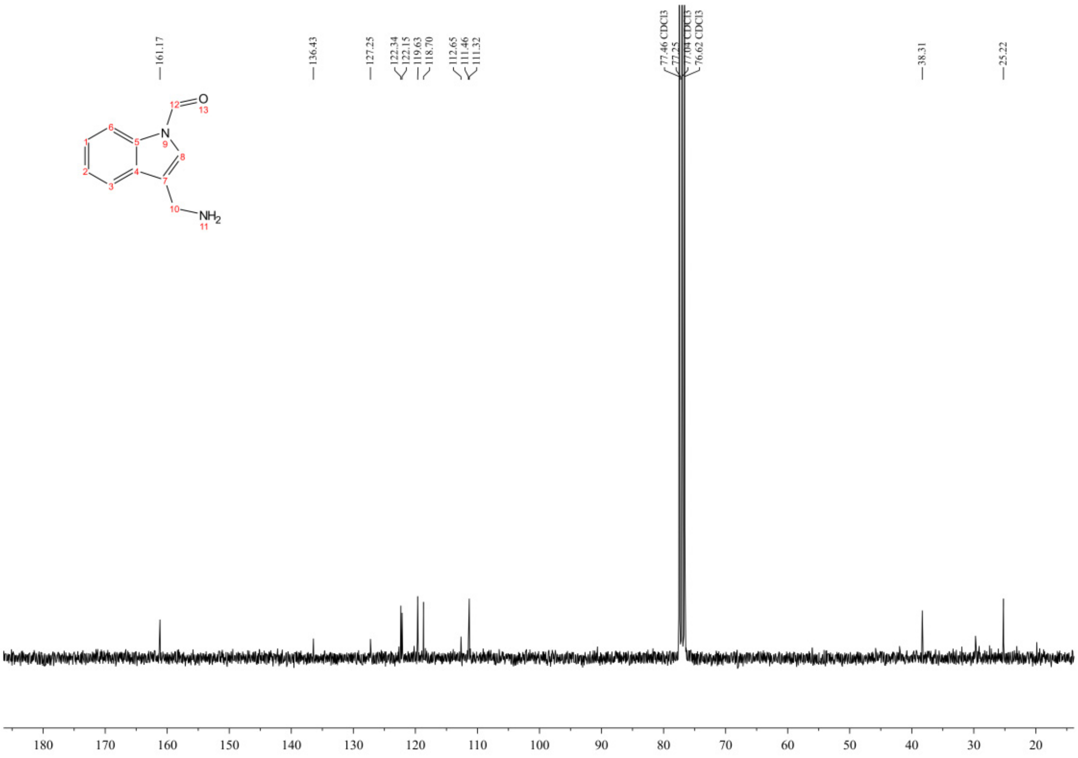

Figure S6. ^{13}C NMR spectrum of indole derivatives A2 ($CDCl_3$, 75MHZ)

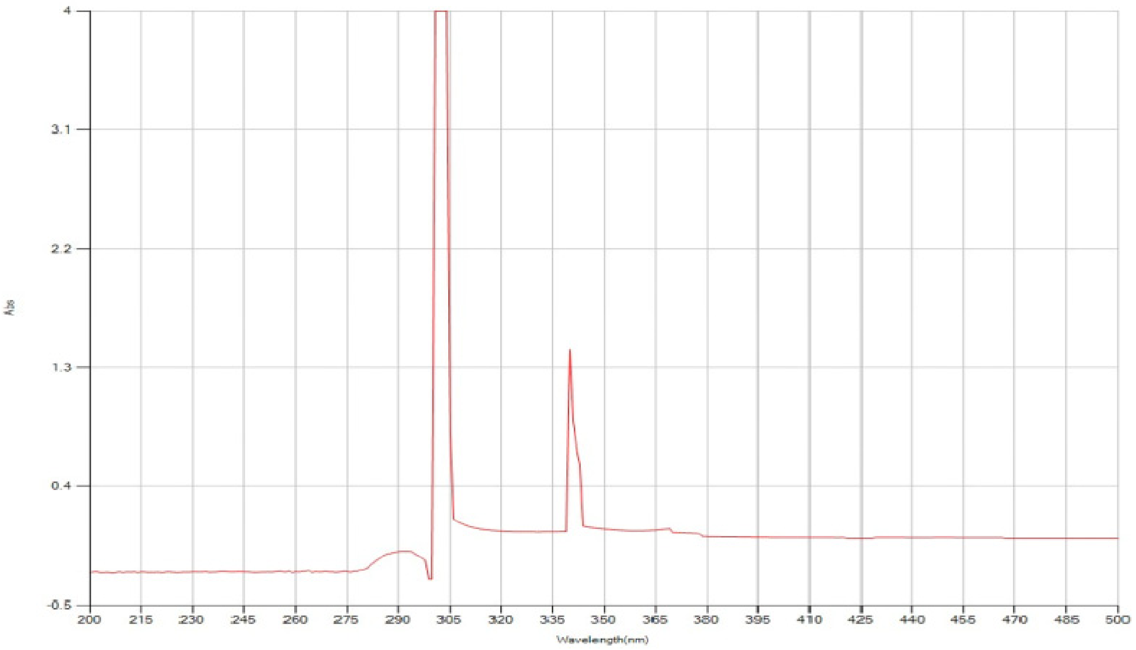

Figure S7. UV spectrum of indole derivatives A2

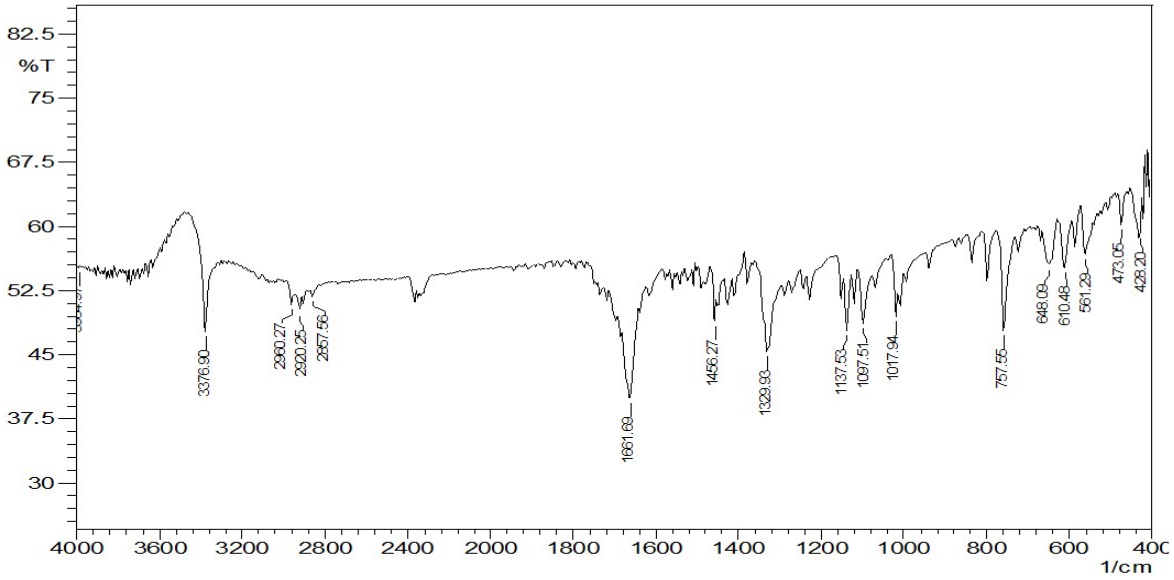

Figure S8. FTIR spectrum of indole derivatives A2

4. Spectrum of indole derivative A3

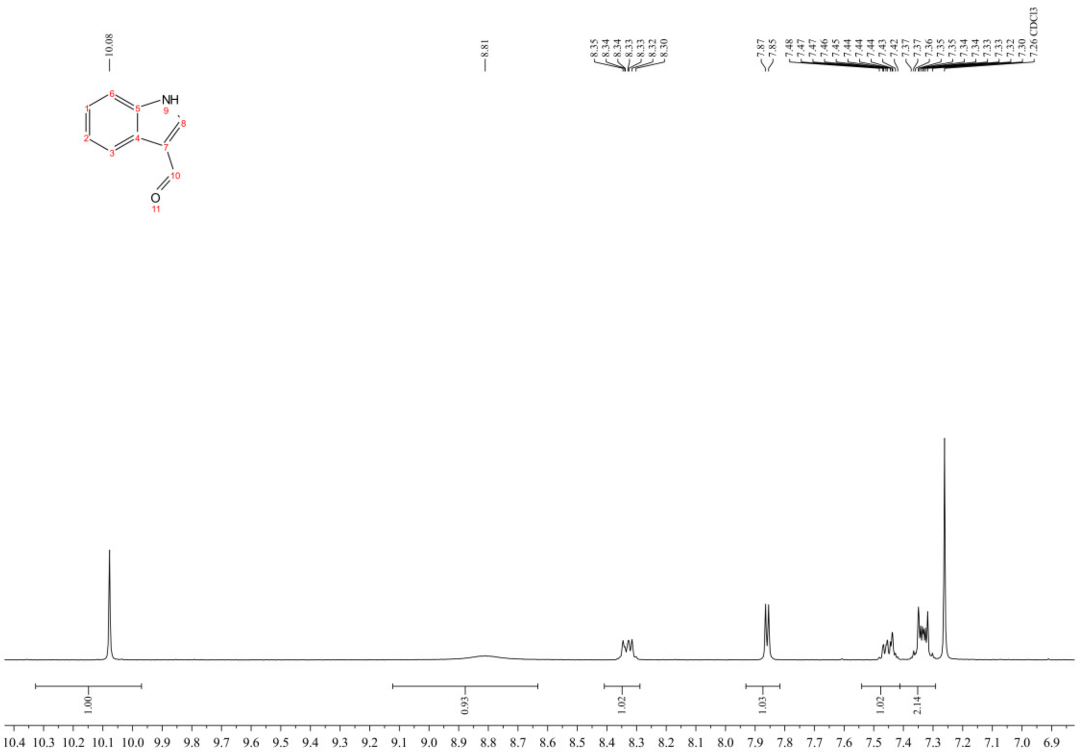

Figure S9. ^{1}H NMR spectrum of indole derivatives A3($CDCl_3$, 300MHZ)

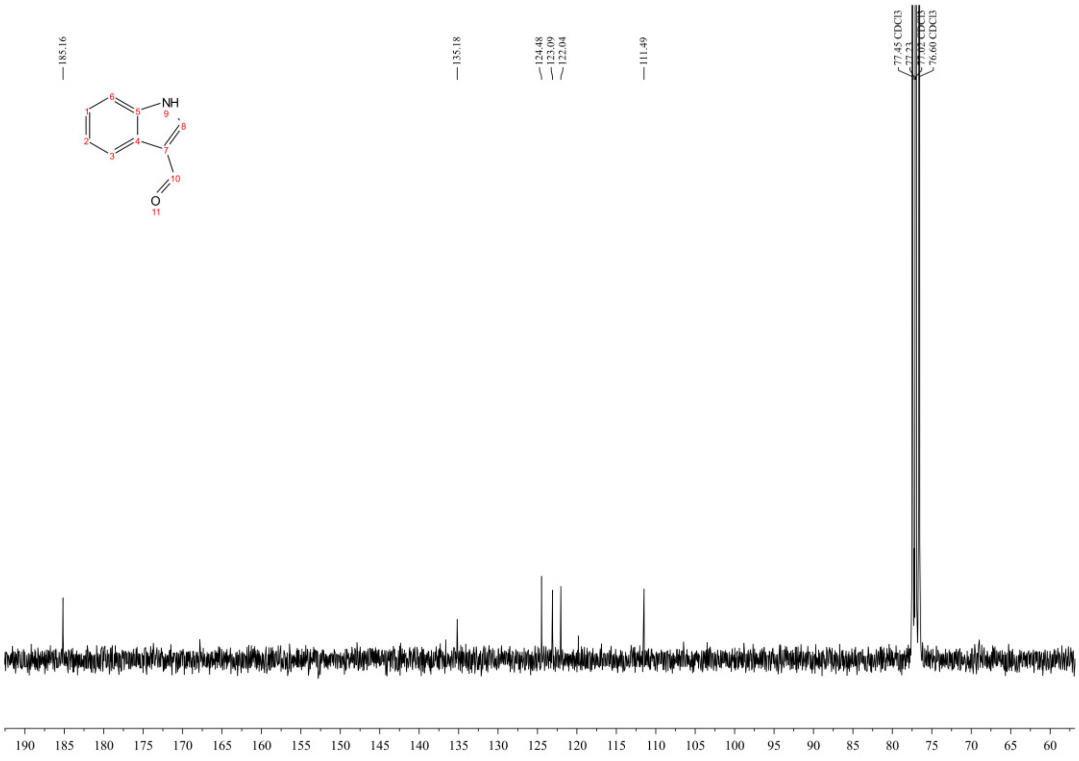

Figure S10. ^{13}C NMR spectrum of indole derivatives A3 ($CDCl_3$, 75MHZ)

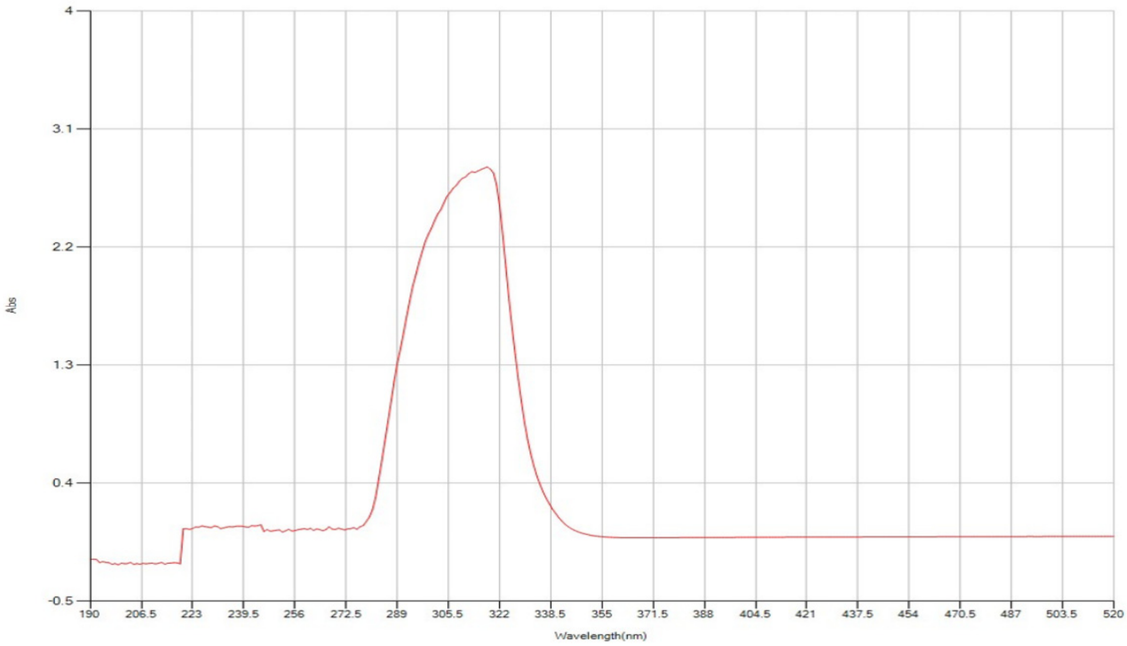

Figure S11. UV spectrum of indole derivatives A2

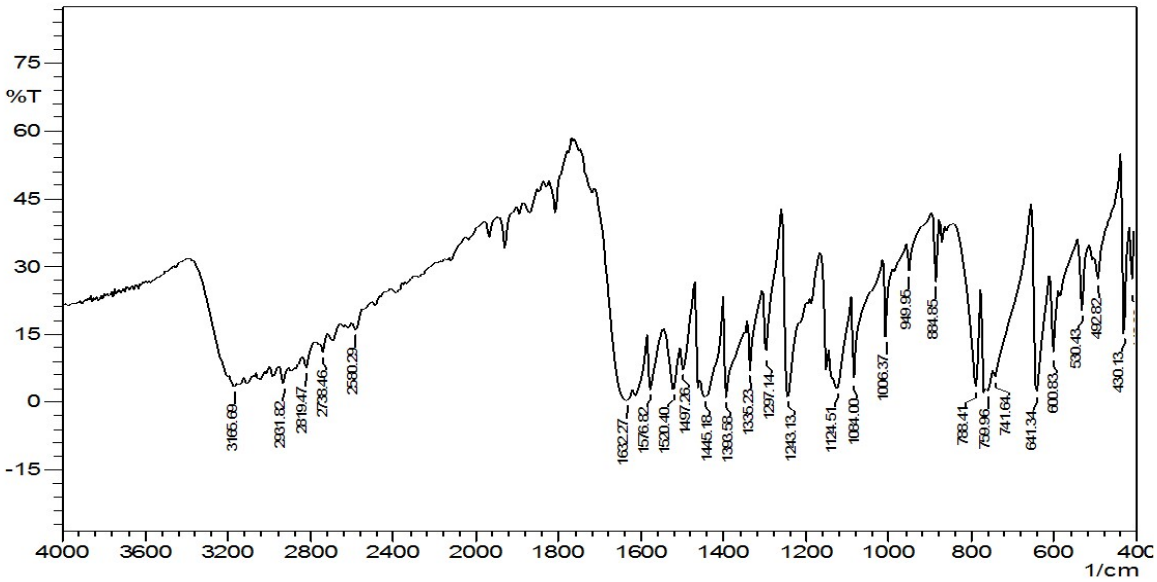

Figure S12. FTIR spectrum of indole derivatives A3

5. Spectrum of indole derivative A4

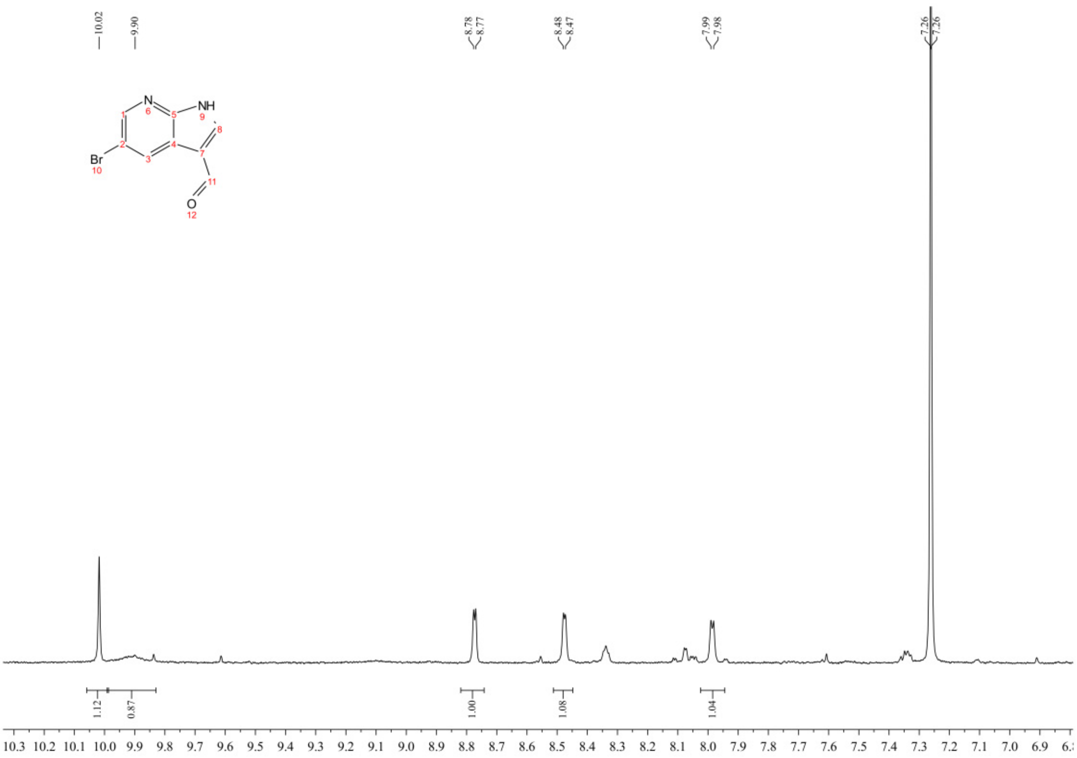

Figure S13. ^{1}H NMR spectrum of indole derivatives A4($CDCl_3$, 300MHZ)

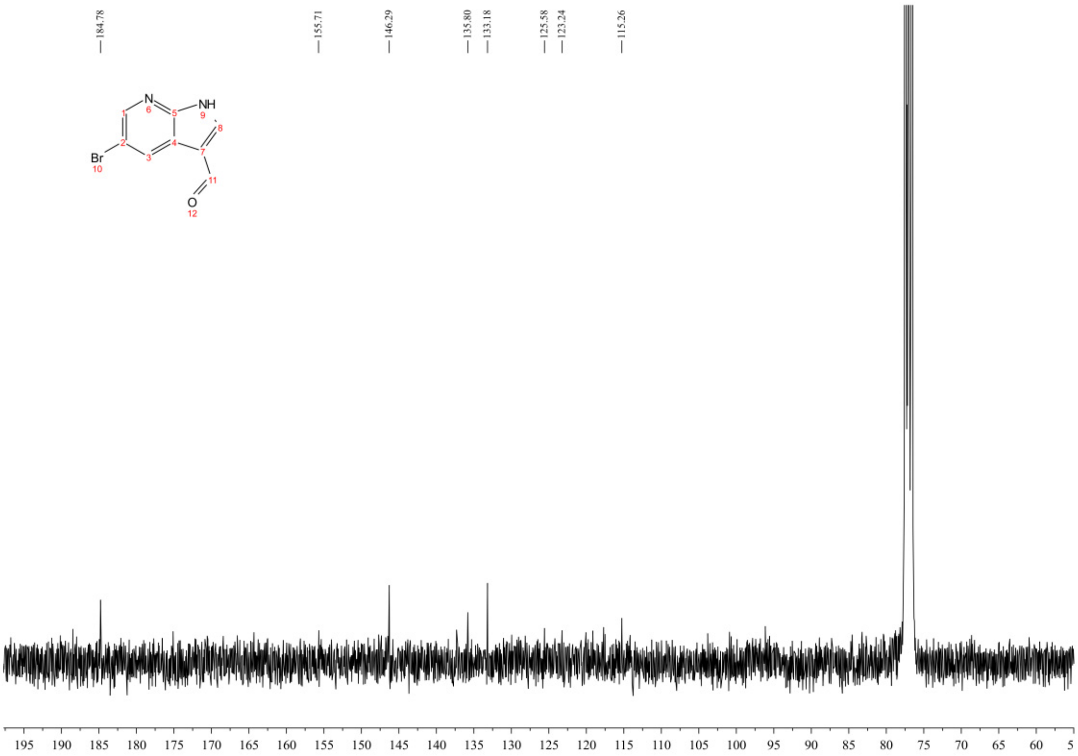

Figure S14. ^{13}C NMR spectrum of indole derivatives A4 ($CDCl_3$, 75MHZ)

6. Spectrum of indole derivative A5

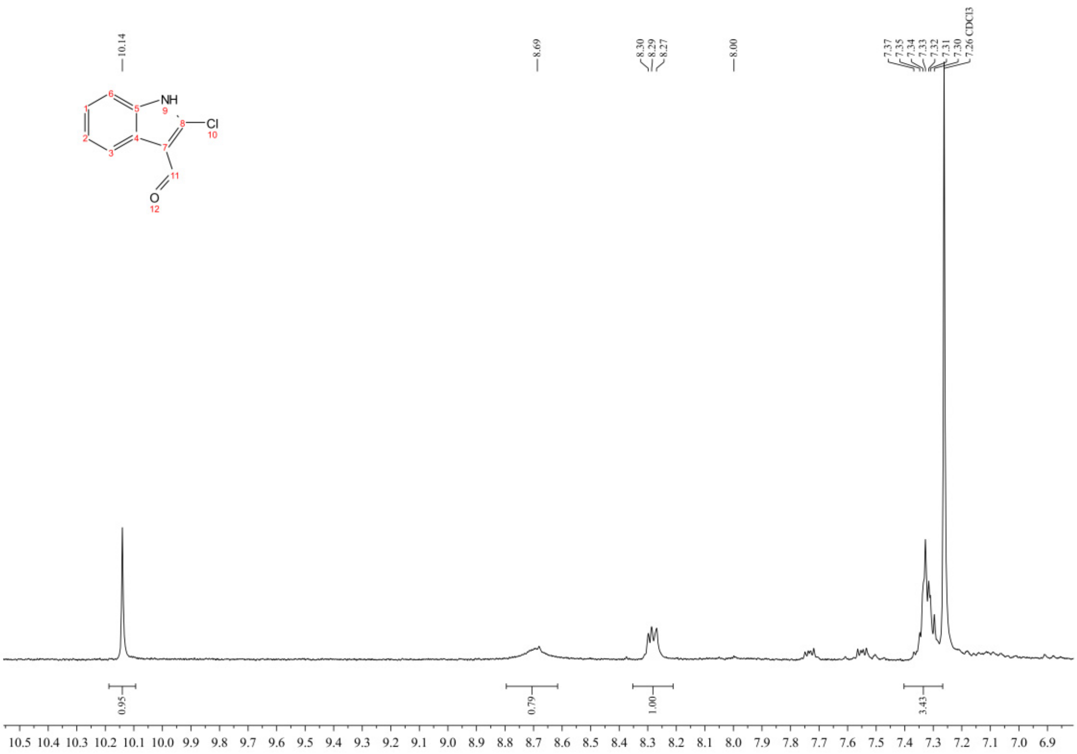

Figure S15. ^{1}H NMR spectrum of indole derivatives A5(CDCl$_3$, 300MHZ)

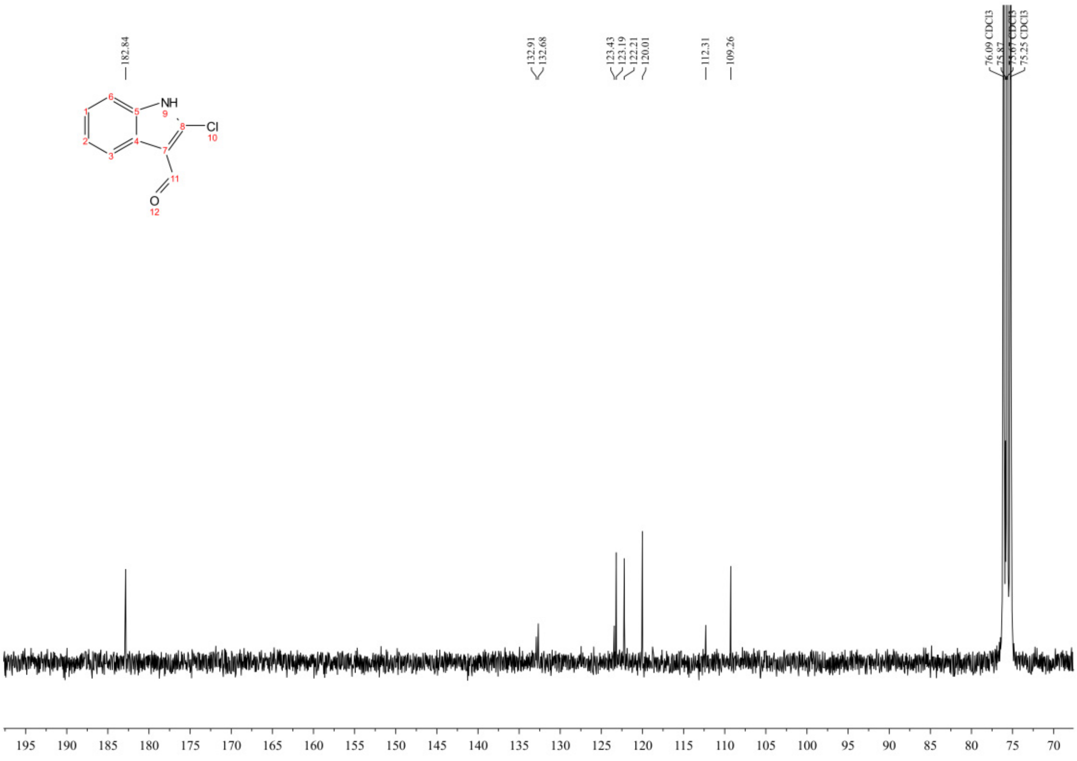

Figure S16. ^{13}C NMR spectrum of indole derivatives A5 (CDCl$_3$, 75MHZ)

7. Spectrum of indole derivative A6

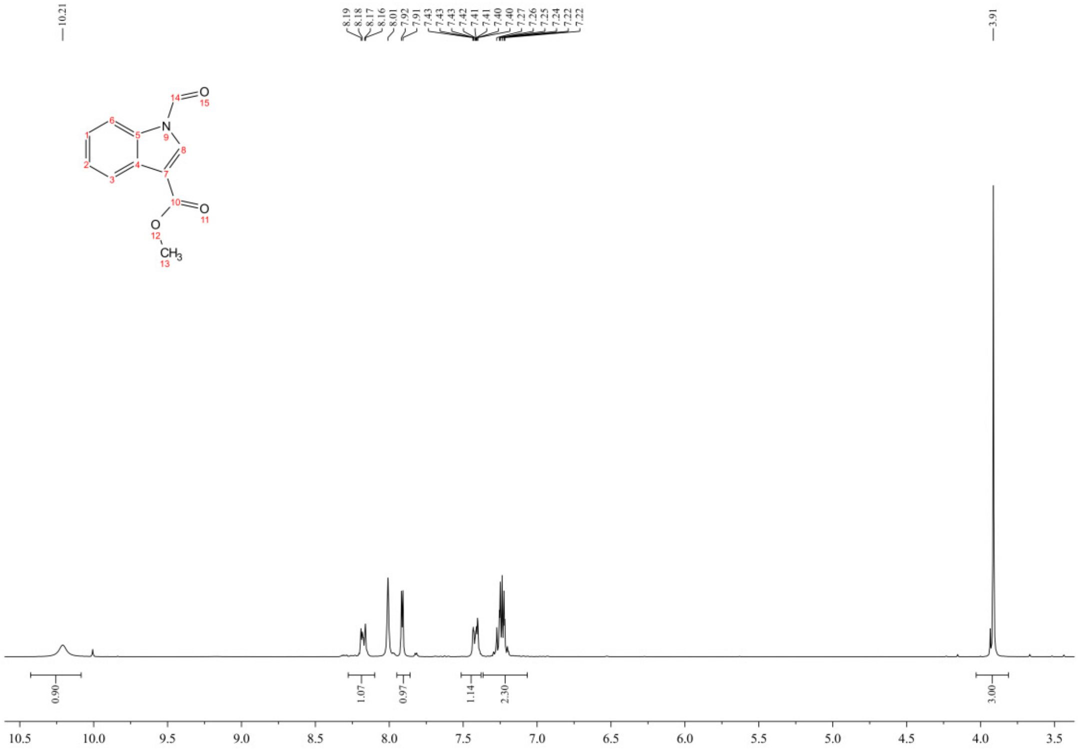

Figure S17. ^{1}H NMR spectrum of indole derivatives A6($CDCl_3$, 300MHZ)

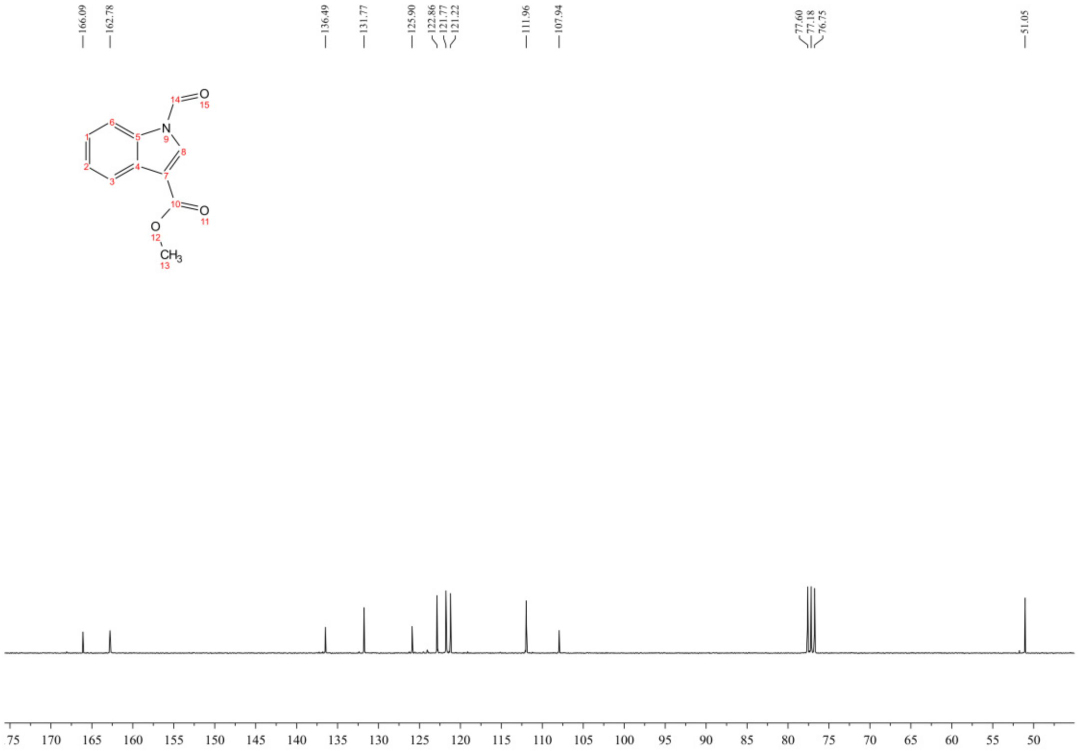

Figure S18. ^{13}C NMR spectrum of indole derivatives A6 ($CDCl_3$, 75MHZ)

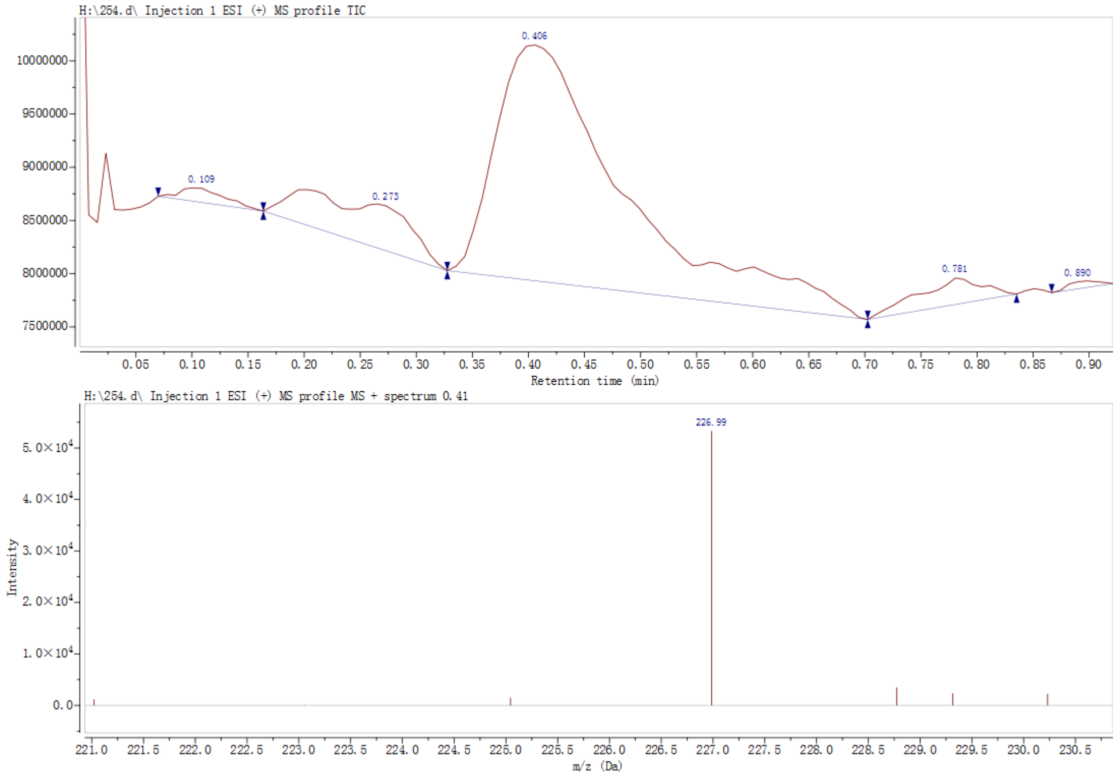

Figure S19. LC-MS-MS spectrum of indole derivatives A6 (ESI(+) Na^{+}+1)

8. Spectrum of indole derivative A7

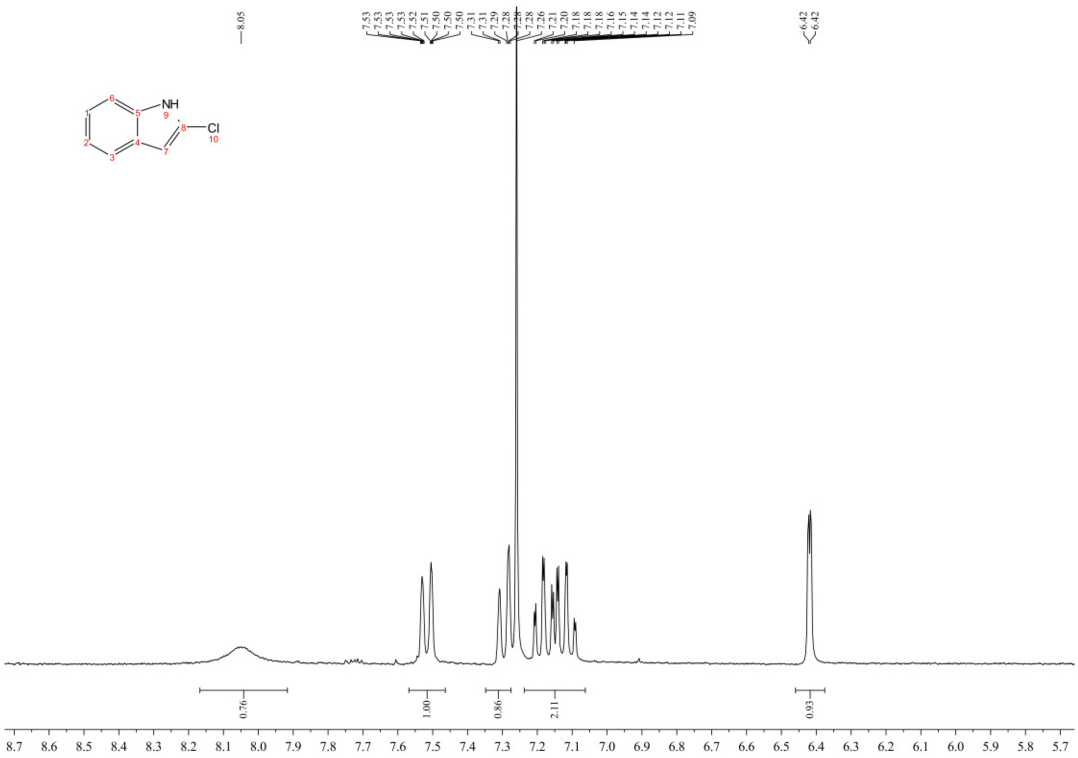

Figure S20. ^{1}H NMR spectrum of indole derivatives A7(CDCl3, 300MHZ)

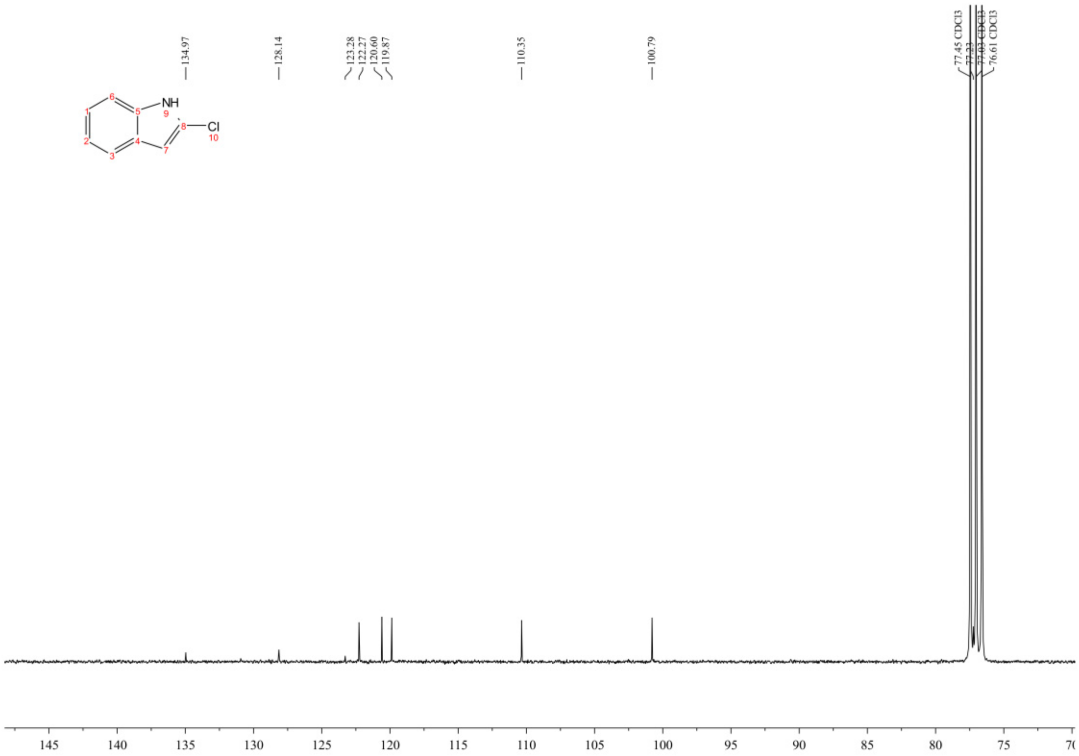

Figure S21. ^{13}C NMR spectrum of indole derivatives A7 (CDCl3, 75MHZ)

9. Spectrum of indole derivative A8

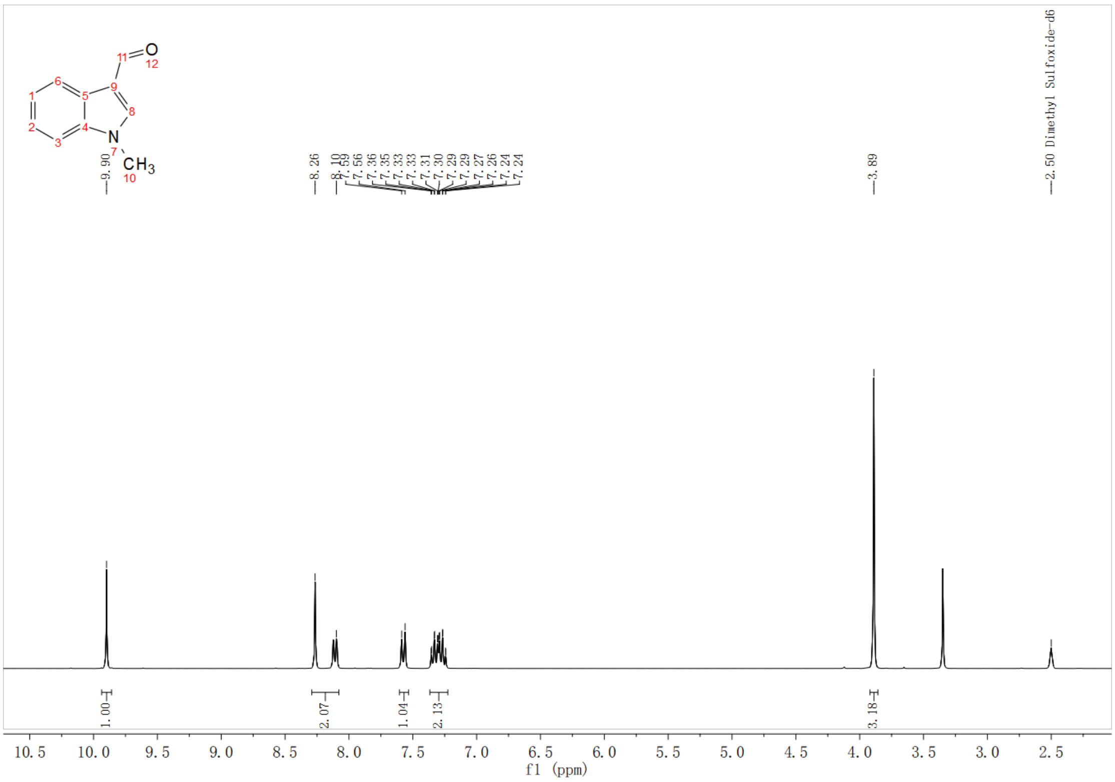

Figure S22. ^{1}H NMR spectrum of indole derivatives A8 (DMSO-d_6, 300MHZ)

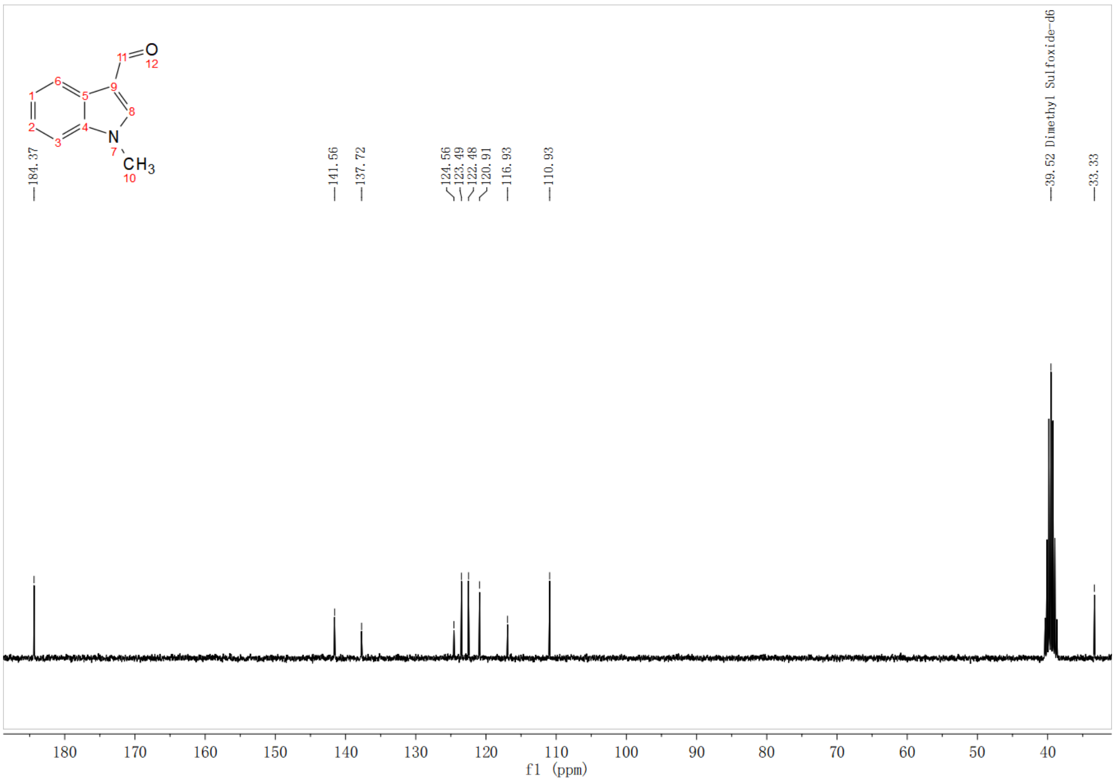

Figure S23. ^{13}C NMR spectrum of indole derivatives A8 (DMSO-d_6, 75MHZ)

10. Spectrum of indole derivative A9

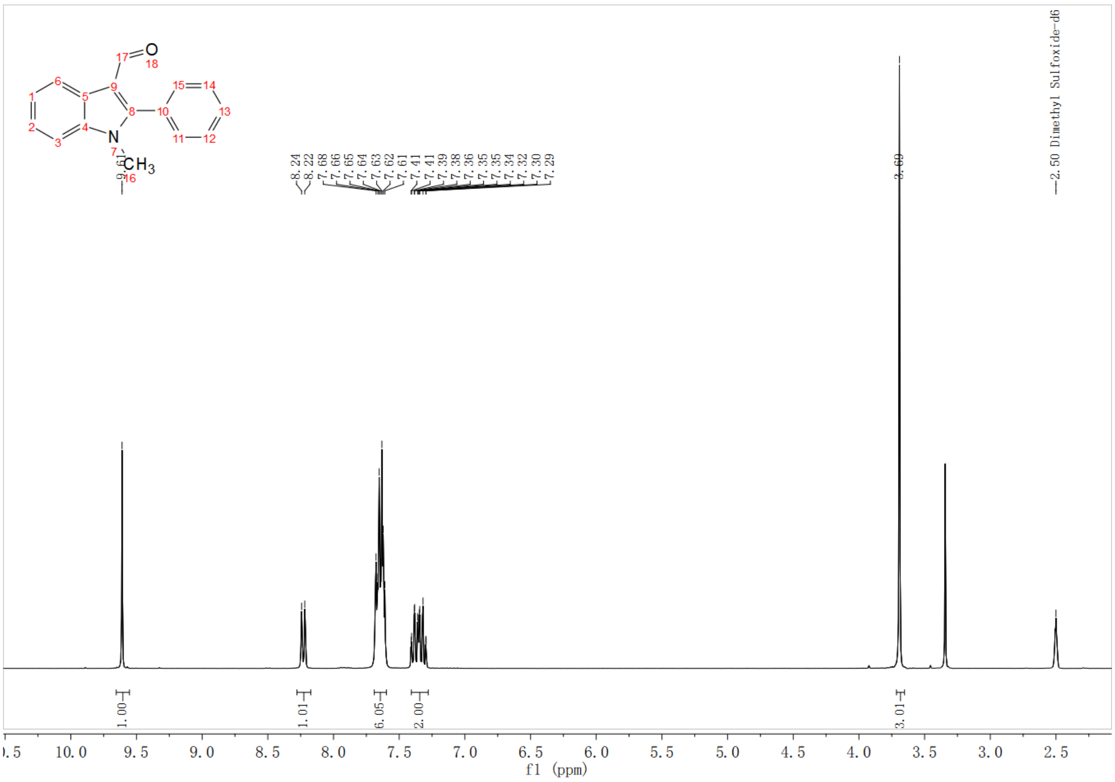

Figure S24. ¹H NMR spectrum of indole derivatives A9 (DMSO-*d*6, 300MHZ)

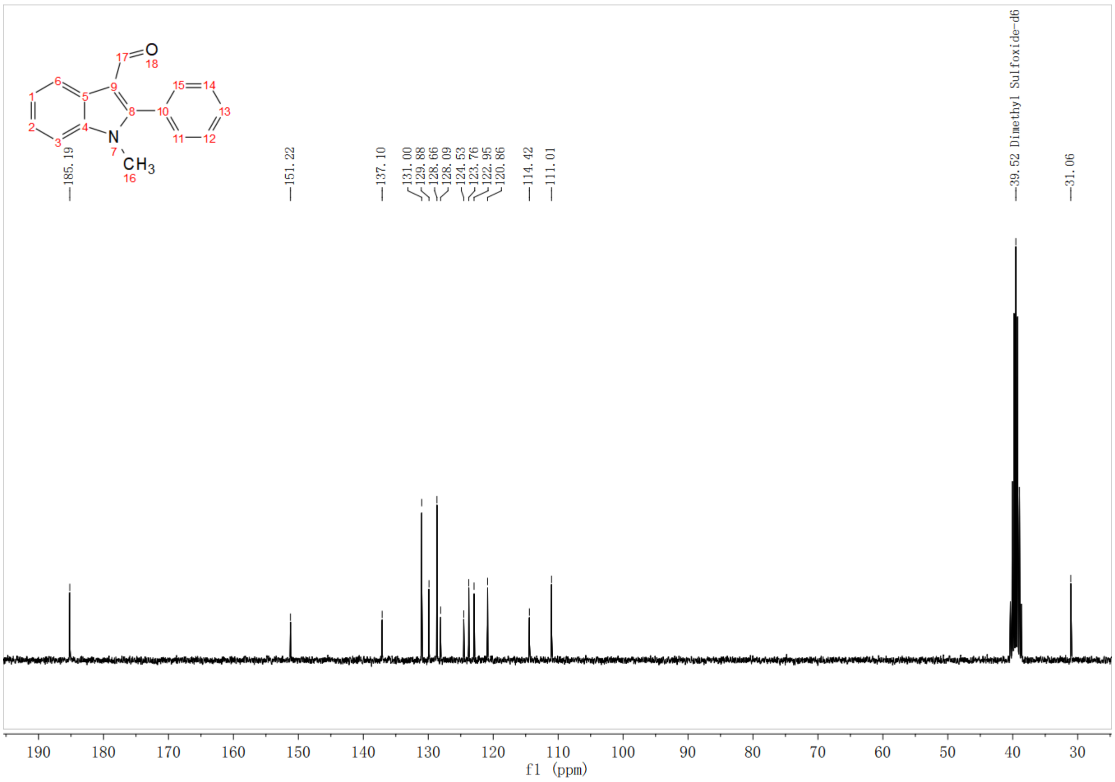

Figure S25. ¹³C NMR spectrum of indole derivatives A9 (DMSO-*d*6, 75MHZ)

11. Pharmacokinetic prediction of compounds

Firstly we imported the structure of the designed drug into Swiss-ADME (SIB Swiss Institute of Bioinformatics, GE, Genève | UNIL University of Lausanne, Vaud, Lausanne，CHE), then exported the SMILE number of the compound and clicked Run to get the predicted pharmacokinetic data of our desired compound. Before performing pharmacological activity experiments, we first need to perform a prediction of the pharmacokinetic properties of our drugs, i.e., their lipophilicity, water solubility, and pharmacokinetics by Swiss-ADME. As shown in Table S1, the designed compounds should have reasonable ranges in the key parameters affecting ADME performance, indicating that these compounds have good drug-like properties. Therefore, the compounds were prepared according to known methods and then subjected to pharmacological activity experiments.

Table S1. The water solubility, lipophilicity and drug-likeness prediction of indole derivatives

Comp.	Water Solubility[1]	Lipophilicity[2]	Drug-likeness[3]
Indole	Soluble<br>Log S:-2.60	Log $P_{o/w}$:1.43	Rule-based filters: yes<br>Bioavailability Score:0.55
A1	Soluble<br>Log S:-2.30	Log $P_{o/w}$:1.48	Rule-based filters: yes<br>Bioavailability Score:0.55
A2	Soluble<br>Log S:-2.05	Log $P_{o/w}$:1.69	Rule-based filters: yes<br>Bioavailability Score:0.55
A3	Soluble<br>Log S:-2.50	Log $P_{o/w}$:1.12	Rule-based filters: yes<br>Bioavailability Score:0.55
A4	Soluble<br>Log S:-2.65	Log $P_{o/w}$:1.36	Rule-based filters: yes<br>Bioavailability Score:0.55
A5	Soluble<br>Log S:-3.00	Log $P_{o/w}$:1.37	Rule-based filters: yes<br>Bioavailability Score:0.55
A6	Soluble<br>Log S:-2.74	Log $P_{o/w}$:1.95	Rule-based filters: yes<br>Bioavailability Score:0.55
A7	Soluble<br>Log S:-3.59	Log $P_{o/w}$:1.77	Rule-based filters: yes<br>Bioavailability Score:0.55
A8	Soluble<br>Log S:-2.41	Log $P_{o/w}$:1.77	Rule-based filters: yes<br>Bioavailability Score:0.55
A9	Soluble<br>Log S:-3.75	Log $P_{o/w}$:2.38	Rule-based filters: yes<br>Bioavailability Score:0.55

[1]**Water Solubility:** *Log S* calculated with ESOL model. Scale: insoluble < -10 < poorly < -6 <moderately < -4 < soluble < -2 < very < 0 <highly. [2] **Lipophilicity:** *Log* $P_{o/w}$ calculated with iLOGP model. [3]**Drug-likeness:** *Rule-based filters*: Lipinski, Ghose, Veber, Egan, Muegge filers. *Bioavailability score*: the probability of a compound to have at least 10% oral bioavailability in rat or measurable Caco-2 permeability. This score defines four classes of compounds with probabilities of 11%, 17%, 56% or 85%.

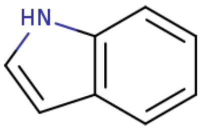

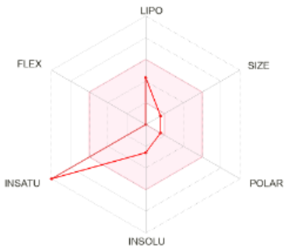

SMILES c1ccc2c(c1)[nH]cc2

Physicochemical Properties	
Formula	C8H7N
Molecular weight	117.15 g/mol
Num. heavy atoms	9
Num. arom. heavy atoms	9
Fraction Csp3	0.00
Num. rotatable bonds	0
Num. H-bond acceptors	0
Num. H-bond donors	1
Molar Refractivity	38.30
TPSA	15.79 Å²

Lipophilicity	
Log $P_{o/w}$ (iLOGP)	1.43
Log $P_{o/w}$ (XLOGP3)	2.05
Log $P_{o/w}$ (WLOGP)	2.17
Log $P_{o/w}$ (MLOGP)	1.57
Log $P_{o/w}$ (SILICOS-IT)	2.66
Consensus Log $P_{o/w}$	1.98

Water Solubility	
Log *S* (ESOL)	-2.60
Solubility	2.96e-01 mg/ml ; 2.52e-03 mol/l
Class	Soluble
Log *S* (Ali)	-2.01
Solubility	1.14e+00 mg/ml ; 9.77e-03 mol/l
Class	Soluble
Log *S* (SILICOS-IT)	-3.23
Solubility	6.90e-02 mg/ml ; 5.89e-04 mol/l
Class	Soluble

Pharmacokinetics	
GI absorption	High
BBB permeant	Yes
P-gp substrate	No
CYP1A2 inhibitor	Yes
CYP2C19 inhibitor	No
CYP2C9 inhibitor	No
CYP2D6 inhibitor	No
CYP3A4 inhibitor	No
Log K_p (skin permeation)	-5.56 cm/s

Druglikeness	
Lipinski	Yes; 0 violation
Ghose	No; 3 violations: MW<160, MR<40, #atoms<20
Veber	Yes
Egan	Yes
Muegge	No; 2 violations: MW<200, Heteroatoms<2
Bioavailability Score	0.55

Medicinal Chemistry	
PAINS	0 alert
Brenk	0 alert
Leadlikeness	No; 1 violation: MW<250
Synthetic accessibility	1.00

Figure S26. Pharmacokinetic prediction of indole

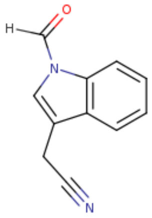

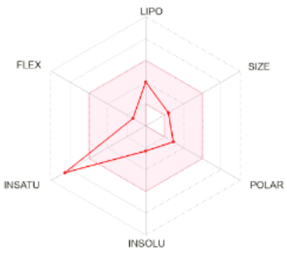

SMILES N#CCc1cn(c2c1cccc2)C=O

Physicochemical Properties	
Formula	C11H8N2O
Molecular weight	184.19 g/mol
Num. heavy atoms	14
Num. arom. heavy atoms	9
Fraction Csp3	0.09
Num. rotatable bonds	2
Num. H-bond acceptors	2
Num. H-bond donors	0
Molar Refractivity	53.31
TPSA	45.79 Å²

Lipophilicity	
Log $P_{o/w}$ (iLOGP)	1.48
Log $P_{o/w}$ (XLOGP3)	1.55
Log $P_{o/w}$ (WLOGP)	2.35
Log $P_{o/w}$ (MLOGP)	1.32
Log $P_{o/w}$ (SILICOS-IT)	1.92
Consensus Log $P_{o/w}$	1.72

Water Solubility	
Log *S* (ESOL)	-2.30
Solubility	9.18e-01 mg/ml ; 4.99e-03 mol/l
Class	Soluble
Log *S* (Ali)	-2.12
Solubility	1.39e+00 mg/ml ; 7.56e-03 mol/l
Class	Soluble
Log *S* (SILICOS-IT)	-2.90
Solubility	2.33e-01 mg/ml ; 1.26e-03 mol/l
Class	Soluble

Pharmacokinetics	
GI absorption	High
BBB permeant	Yes
P-gp substrate	No
CYP1A2 inhibitor	Yes
CYP2C19 inhibitor	No
CYP2C9 inhibitor	No
CYP2D6 inhibitor	No
CYP3A4 inhibitor	No
Log K_p (skin permeation)	-6.32 cm/s

Druglikeness	
Lipinski	Yes; 0 violation
Ghose	Yes
Veber	Yes
Egan	Yes
Muegge	No; 1 violation: MW<200
Bioavailability Score	0.55

Medicinal Chemistry	
PAINS	0 alert
Brenk	1 alert: aldehyde
Leadlikeness	No; 1 violation: MW<250
Synthetic accessibility	1.62

Figure S27. Pharmacokinetic prediction of A1

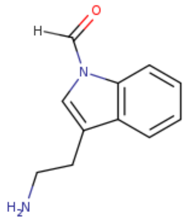

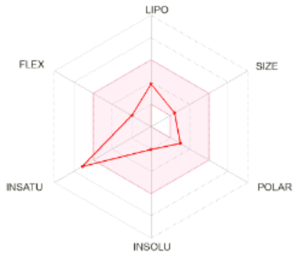

SMILES NCCc1cn(c2c1cccc2)C=O

Physicochemical Properties	
Formula	C11H12N2O
Molecular weight	188.23 g/mol
Num. heavy atoms	14
Num. arom. heavy atoms	9
Fraction Csp3	0.18
Num. rotatable bonds	3
Num. H-bond acceptors	2
Num. H-bond donors	1
Molar Refractivity	56.27
TPSA	48.02 Å²
**Lipophilicity**	
Log $P_{o/w}$ (iLOGP)	1.69
Log $P_{o/w}$ (XLOGP3)	1.22
Log $P_{o/w}$ (WLOGP)	1.78
Log $P_{o/w}$ (MLOGP)	1.40
Log $P_{o/w}$ (SILICOS-IT)	1.56
Consensus Log $P_{o/w}$	1.53

Water Solubility	
Log *S* (ESOL)	-2.05
Solubility	1.66e+00 mg/ml ; 8.84e-03 mol/l
Class	Soluble
Log *S* (Ali)	-1.83
Solubility	2.81e+00 mg/ml ; 1.49e-02 mol/l
Class	Very soluble
Log *S* (SILICOS-IT)	-2.86
Solubility	2.60e-01 mg/ml ; 1.38e-03 mol/l
Class	Soluble
**Pharmacokinetics**	
GI absorption	High
BBB permeant	Yes
P-gp substrate	No
CYP1A2 inhibitor	No
CYP2C19 inhibitor	No
CYP2C9 inhibitor	No
CYP2D6 inhibitor	No
CYP3A4 inhibitor	No
Log K_p (skin permeation)	-6.58 cm/s
**Druglikeness**	
Lipinski	Yes; 0 violation
Ghose	Yes
Veber	Yes
Egan	Yes
Muegge	No; 1 violation: MW<200
Bioavailability Score	0.55
**Medicinal Chemistry**	
PAINS	0 alert
Brenk	1 alert: aldehyde
Leadlikeness	No; 1 violation: MW<250
Synthetic accessibility	1.72

Figure S28. Pharmacokinetic prediction of A2

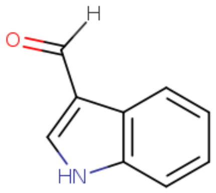

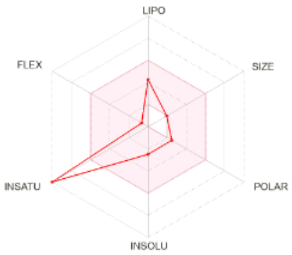

SMILES O=Cc1c[nH]c2c1cccc2

Physicochemical Properties	
Formula	C9H7NO
Molecular weight	145.16 g/mol
Num. heavy atoms	11
Num. arom. heavy atoms	9
Fraction Csp3	0.00
Num. rotatable bonds	1
Num. H-bond acceptors	1
Num. H-bond donors	1
Molar Refractivity	43.69
TPSA	32.86 Å²
**Lipophilicity**	
Log $P_{o/w}$ (iLOGP)	1.12
Log $P_{o/w}$ (XLOGP3)	1.93
Log $P_{o/w}$ (WLOGP)	1.98
Log $P_{o/w}$ (MLOGP)	0.88
Log $P_{o/w}$ (SILICOS-IT)	2.69

Water Solubility	
Log *S* (ESOL)	-2.50
Solubility	4.64e-01 mg/ml ; 3.20e-03 mol/l
Class	Soluble
Log *S* (Ali)	-2.24
Solubility	8.28e-01 mg/ml ; 5.70e-03 mol/l
Class	Soluble
Log *S* (SILICOS-IT)	-3.21
Solubility	8.97e-02 mg/ml ; 6.18e-04 mol/l
Class	Soluble
**Pharmacokinetics**	
GI absorption	High
BBB permeant	Yes
P-gp substrate	No
CYP1A2 inhibitor	Yes
CYP2C19 inhibitor	No
CYP2C9 inhibitor	No
CYP2D6 inhibitor	No
CYP3A4 inhibitor	No
Log K_p (skin permeation)	-5.82 cm/s
**Druglikeness**	
Lipinski	Yes; 0 violation
Ghose	No; 2 violations: MW<160, #atoms<20
Veber	Yes
Egan	Yes
Muegge	No; 1 violation: MW<200
Bioavailability Score	0.55
**Medicinal Chemistry**	
PAINS	0 alert

Figure S29. Pharmacokinetic prediction of A3

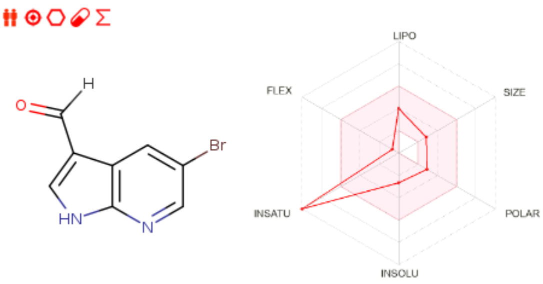

SMILES Brc1cc2c(C=O)c[nH]c2nc1

Physicochemical Properties	
Formula	C8H5BrN2O
Molecular weight	225.04 g/mol
Num. heavy atoms	12
Num. arom. heavy atoms	9
Fraction Csp3	0.00
Num. rotatable bonds	1
Num. H-bond acceptors	2
Num. H-bond donors	1
Molar Refractivity	49.18
TPSA	45.75 Å²

Lipophilicity	
Log $P_{o/w}$ (iLOGP)	1.36
Log $P_{o/w}$ (XLOGP3)	1.47
Log $P_{o/w}$ (WLOGP)	2.14
Log $P_{o/w}$ (MLOGP)	1.09
Log $P_{o/w}$ (SILICOS-IT)	2.82
Consensus Log $P_{o/w}$	1.78

Water Solubility	
Log *S* (ESOL)	-2.65
Solubility	5.03e-01 mg/ml ; 2.24e-03 mol/l
Class	Soluble
Log *S* (Ali)	-2.04
Solubility	2.06e+00 mg/ml ; 9.18e-03 mol/l
Class	Soluble
Log *S* (SILICOS-IT)	-3.72
Solubility	4.30e-02 mg/ml ; 1.91e-04 mol/l
Class	Soluble

Pharmacokinetics	
GI absorption	High
BBB permeant	Yes
P-gp substrate	No
CYP1A2 inhibitor	Yes
CYP2C19 inhibitor	No
CYP2C9 inhibitor	No
CYP2D6 inhibitor	No
CYP3A4 inhibitor	No
Log K_p (skin permeation)	-6.63 cm/s

Druglikeness	
Lipinski	Yes; 0 violation
Ghose	No; 1 violation: #atoms<20
Veber	Yes
Egan	Yes
Muegge	Yes
Bioavailability Score	0.55

Medicinal Chemistry	
PAINS	0 alert
Brenk	1 alert: aldehyde
Leadlikeness	No; 1 violation: MW<250
Synthetic accessibility	1.43

Figure S30. Pharmacokinetic prediction of A4

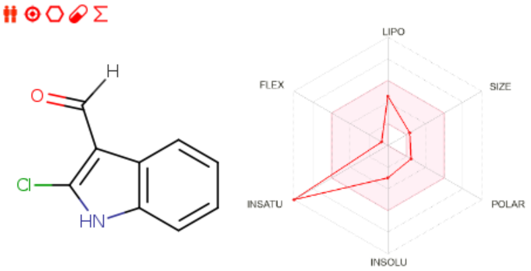

SMILES O=Cc1c(Cl)[nH]c2c1cccc2

Physicochemical Properties	
Formula	C9H6ClNO
Molecular weight	179.60 g/mol
Num. heavy atoms	12
Num. arom. heavy atoms	9
Fraction Csp3	0.00
Num. rotatable bonds	1
Num. H-bond acceptors	1
Num. H-bond donors	1
Molar Refractivity	48.70
TPSA	32.86 Å²

Lipophilicity	
Log $P_{o/w}$ (iLOGP)	1.37
Log $P_{o/w}$ (XLOGP3)	2.47
Log $P_{o/w}$ (WLOGP)	2.63
Log $P_{o/w}$ (MLOGP)	1.45
Log $P_{o/w}$ (SILICOS-IT)	3.29
Consensus Log $P_{o/w}$	2.24

Water Solubility	
Log *S* (ESOL)	-3.00
Solubility	1.80e-01 mg/ml ; 1.00e-03 mol/l
Class	Soluble
Log *S* (Ali)	-2.80
Solubility	2.82e-01 mg/ml ; 1.57e-03 mol/l
Class	Soluble
Log *S* (SILICOS-IT)	-3.85
Solubility	2.56e-02 mg/ml ; 1.43e-04 mol/l
Class	Soluble

Pharmacokinetics	
GI absorption	High
BBB permeant	Yes
P-gp substrate	No
CYP1A2 inhibitor	Yes
CYP2C19 inhibitor	No
CYP2C9 inhibitor	No
CYP2D6 inhibitor	No
CYP3A4 inhibitor	No
Log K_p (skin permeation)	-5.64 cm/s

Druglikeness	
Lipinski	Yes; 0 violation
Ghose	No; 1 violation: #atoms<20
Veber	Yes
Egan	Yes
Muegge	No; 1 violation: MW<200
Bioavailability Score	0.55

Medicinal Chemistry	
PAINS	0 alert
Brenk	1 alert: aldehyde
Leadlikeness	No; 1 violation: MW<250
Synthetic accessibility	1.41

Figure S31. Pharmacokinetic prediction of A5

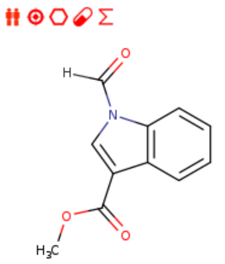

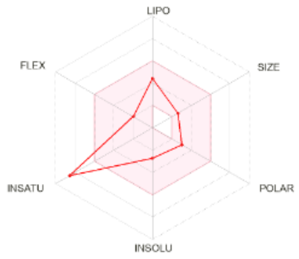

SMILES COC(=O)c1cn(c2c1cccc2)C=O

Physicochemical Properties	
Formula	C11H9NO3
Molecular weight	203.19 g/mol
Num. heavy atoms	15
Num. arom. heavy atoms	9
Fraction Csp3	0.09
Num. rotatable bonds	3
Num. H-bond acceptors	3
Num. H-bond donors	0
Molar Refractivity	55.07
TPSA	48.30 Å²

Lipophilicity	
Log $P_{o/w}$ (iLOGP)	1.95
Log $P_{o/w}$ (XLOGP3)	2.22
Log $P_{o/w}$ (WLOGP)	2.07
Log $P_{o/w}$ (MLOGP)	1.55
Log $P_{o/w}$ (SILICOS-IT)	1.48
Consensus Log $P_{o/w}$	1.85

Water Solubility	
Log *S* (ESOL)	-2.74
Solubility	3.66e-01 mg/ml ; 1.80e-03 mol/l
Class	Soluble
Log *S* (Ali)	-2.87
Solubility	2.75e-01 mg/ml ; 1.35e-03 mol/l
Class	Soluble
Log *S* (SILICOS-IT)	-2.49
Solubility	6.59e-01 mg/ml ; 3.24e-03 mol/l
Class	Soluble

Pharmacokinetics	
GI absorption	High
BBB permeant	Yes
P-gp substrate	No
CYP1A2 inhibitor	Yes
CYP2C19 inhibitor	No
CYP2C9 inhibitor	No
CYP2D6 inhibitor	No
CYP3A4 inhibitor	No
Log K_p (skin permeation)	-5.96 cm/s

Druglikeness	
Lipinski	Yes; 0 violation
Ghose	Yes
Veber	Yes
Egan	Yes
Muegge	Yes
Bioavailability Score	0.55

Medicinal Chemistry	
PAINS	0 alert
Brenk	1 alert: aldehyde
Leadlikeness	No; 1 violation: MW<250
Synthetic accessibility	1.68

Figure S32. Pharmacokinetic prediction of A6

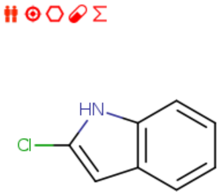

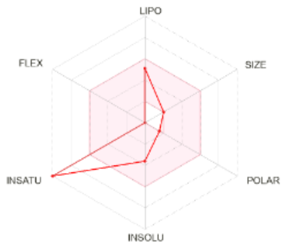

SMILES Clc1cc2c([nH]1)cccc2

Physicochemical Properties	
Formula	C8H6ClN
Molecular weight	151.59 g/mol
Num. heavy atoms	10
Num. arom. heavy atoms	9
Fraction Csp3	0.00
Num. rotatable bonds	0
Num. H-bond acceptors	0
Num. H-bond donors	1
Molar Refractivity	43.31
TPSA	15.79 Å²

Lipophilicity	
Log $P_{o/w}$ (iLOGP)	1.77
Log $P_{o/w}$ (XLOGP3)	3.41
Log $P_{o/w}$ (WLOGP)	2.82
Log $P_{o/w}$ (MLOGP)	2.16
Log $P_{o/w}$ (SILICOS-IT)	3.22
Consensus Log $P_{o/w}$	2.67

Water Solubility	
Log *S* (ESOL)	-3.59
Solubility	3.86e-02 mg/ml ; 2.55e-04 mol/l
Class	Soluble
Log *S* (Ali)	-3.42
Solubility	5.75e-02 mg/ml ; 3.79e-04 mol/l
Class	Soluble
Log *S* (SILICOS-IT)	-3.88
Solubility	1.99e-02 mg/ml ; 1.32e-04 mol/l
Class	Soluble

Pharmacokinetics	
GI absorption	High
BBB permeant	Yes
P-gp substrate	No
CYP1A2 inhibitor	Yes
CYP2C19 inhibitor	No
CYP2C9 inhibitor	No
CYP2D6 inhibitor	No
CYP3A4 inhibitor	No
Log K_p (skin permeation)	-4.80 cm/s

Druglikeness	
Lipinski	Yes; 0 violation
Ghose	No; 2 violations: MW<160, #atoms<20
Veber	Yes
Egan	Yes
Muegge	No; 2 violations: MW<200, Heteroatoms<2
Bioavailability Score	0.55

Medicinal Chemistry	
PAINS	0 alert
Brenk	0 alert
Leadlikeness	No; 1 violation: MW<250
Synthetic accessibility	1.37

Figure S33. Pharmacokinetic prediction of A7

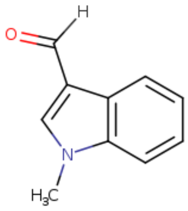

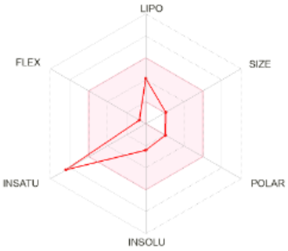

SMILES O=Cc1cn(c2c1cccc2)C

Physicochemical Properties	
Formula	C10H9NO
Molecular weight	159.18 g/mol
Num. heavy atoms	12
Num. arom. heavy atoms	9
Fraction Csp3	0.10
Num. rotatable bonds	1
Num. H-bond acceptors	1
Num. H-bond donors	0
Molar Refractivity	48.59
TPSA	22.00 Å²

Lipophilicity	
Log $P_{o/w}$ (iLOGP)	1.71
Log $P_{o/w}$ (XLOGP3)	1.73
Log $P_{o/w}$ (WLOGP)	1.99
Log $P_{o/w}$ (MLOGP)	1.18
Log $P_{o/w}$ (SILICOS-IT)	2.09
Consensus Log $P_{o/w}$	1.74

Water Solubility	
Log *S* (ESOL)	-2.41
Solubility	6.25e-01 mg/ml ; 3.93e-03 mol/l
Class	Soluble
Log *S* (Ali)	-1.81
Solubility	2.47e+00 mg/ml ; 1.55e-02 mol/l
Class	Very soluble
Log *S* (SILICOS-IT)	-2.78
Solubility	2.64e-01 mg/ml ; 1.66e-03 mol/l
Class	Soluble

Pharmacokinetics	
GI absorption	High
BBB permeant	Yes
P-gp substrate	No
CYP1A2 inhibitor	Yes
CYP2C19 inhibitor	No
CYP2C9 inhibitor	No
CYP2D6 inhibitor	No
CYP3A4 inhibitor	No
Log K_p (skin permeation)	-6.04 cm/s

Druglikeness	
Lipinski	Yes; 0 violation
Ghose	No; 1 violation: MW<160
Veber	Yes
Egan	Yes
Muegge	No; 1 violation: MW<200
Bioavailability Score	0.55

Medicinal Chemistry	
PAINS	0 alert
Brenk	1 alert: aldehyde
Leadlikeness	No; 1 violation: MW<250
Synthetic accessibility	1.11

Figure S34. Pharmacokinetic prediction of A8

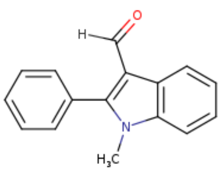

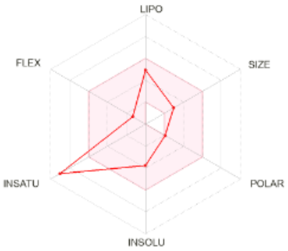

SMILES O=Cc1c(c2ccccc2)n(c2c1cccc2)C

Physicochemical Properties	
Formula	C16H13NO
Molecular weight	235.28 g/mol
Num. heavy atoms	18
Num. arom. heavy atoms	15
Fraction Csp3	0.06
Num. rotatable bonds	2
Num. H-bond acceptors	1
Num. H-bond donors	0
Molar Refractivity	74.02
TPSA	22.00 Å²

Lipophilicity	
Log $P_{o/w}$ (iLOGP)	2.38
Log $P_{o/w}$ (XLOGP3)	3.12
Log $P_{o/w}$ (WLOGP)	3.66
Log $P_{o/w}$ (MLOGP)	2.56
Log $P_{o/w}$ (SILICOS-IT)	3.72
Consensus Log $P_{o/w}$	3.09

Water Solubility	
Log *S* (ESOL)	-3.75
Solubility	4.19e-02 mg/ml ; 1.78e-04 mol/l
Class	Soluble
Log *S* (Ali)	-3.25
Solubility	1.32e-01 mg/ml ; 5.61e-04 mol/l
Class	Soluble
Log *S* (SILICOS-IT)	-5.32
Solubility	1.13e-03 mg/ml ; 4.81e-06 mol/l
Class	Moderately soluble

Pharmacokinetics	
GI absorption	High
BBB permeant	Yes
P-gp substrate	No
CYP1A2 inhibitor	Yes
CYP2C19 inhibitor	Yes
CYP2C9 inhibitor	No
CYP2D6 inhibitor	Yes
CYP3A4 inhibitor	No
Log K_p (skin permeation)	-5.52 cm/s

Druglikeness	
Lipinski	Yes; 0 violation
Ghose	Yes
Veber	Yes
Egan	Yes
Muegge	Yes
Bioavailability Score	0.55

Medicinal Chemistry	
PAINS	0 alert
Brenk	1 alert: aldehyde
Leadlikeness	No; 1 violation: MW<250
Synthetic accessibility	2.01

Figure S35. Pharmacokinetic prediction of A9
